# Supplementary material for: Damaging missense variants in IGF1R implicate a role for IGF-1 resistance in the etiology of type 2 diabetes
Source: Cell Genom. 2022 Nov 7;2(12):100208. doi: 10.1016/j.xgen.2022.100208 (PMC9750938; doi:10.1016/j.xgen.2022.100208)
Supplement: Document S1. Figures S1–S4 [file mmc1.pdf]

Cell Genomics, Volume *2*

Supplemental information

Damaging missense variants in *IGF1R* implicate a role for IGF-1 resistance in the etiology of type 2 diabetes

Eugene J. Gardner, Katherine A. Kentistou, Stasa Stankovic, Samuel Lockhart, Eleanor Wheeler, Felix R. Day, Nicola D. Kerrison, Nicholas J. Wareham, Claudia Langenberg, Stephen O'Rahilly, Ken K. Ong, and John R.B. Perry

Supplementary Materials for

Damaging missense variants in *IGF1R* implicate a role for IGF-1 resistance in the aetiology of type 2 diabetes

Eugene J. Gardner[@], Katherine Kentistou, Stasa Stankovic, Samuel Lockhart, Eleanor Wheeler, Felix R. Day, Nicola D. Kerrison, Nicholas J. Wareham, Claudia Langenberg, Stephen O'Rahilly*, Ken K. Ong*, John R. B. Perry*,[@]

* These authors jointly supervised this work
[@] Corresponding authors: eugene.gardner@mrc-epid.cam.ac.uk; john.perry@mrc-epid.cam.ac.uk

Supplementary Figures

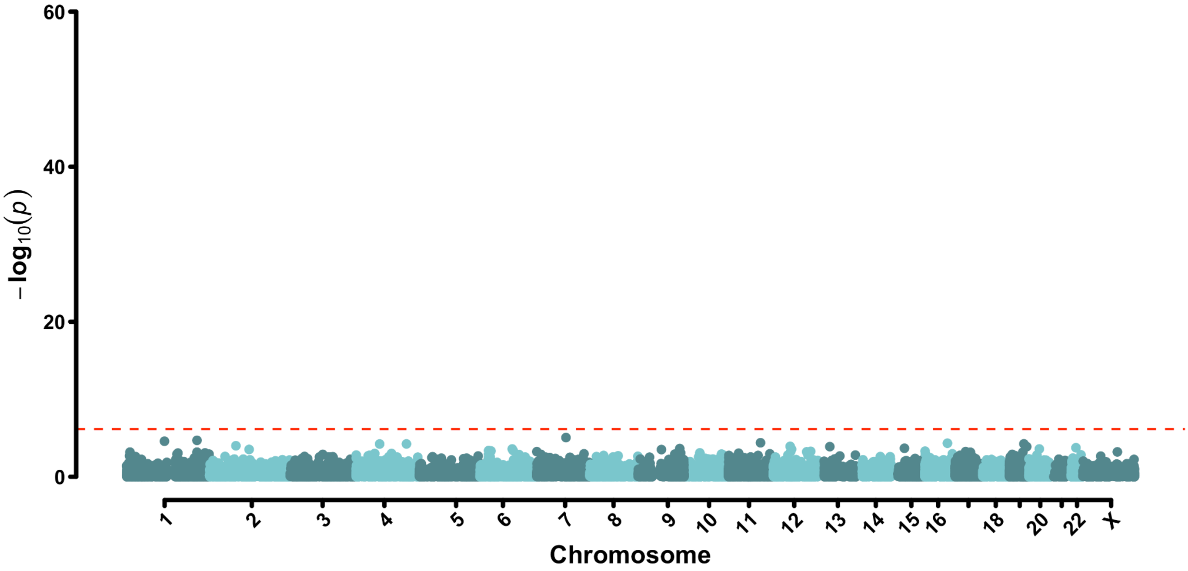

Supplementary Figure S1. Exome-wide association results for synonymous variants, related to Figure 1.

Plotted are per-gene burden results when only considering synonymous variants. The red line indicates our exome-wide significant p value after Bonferroni correction of 6.9×10^{-7}.

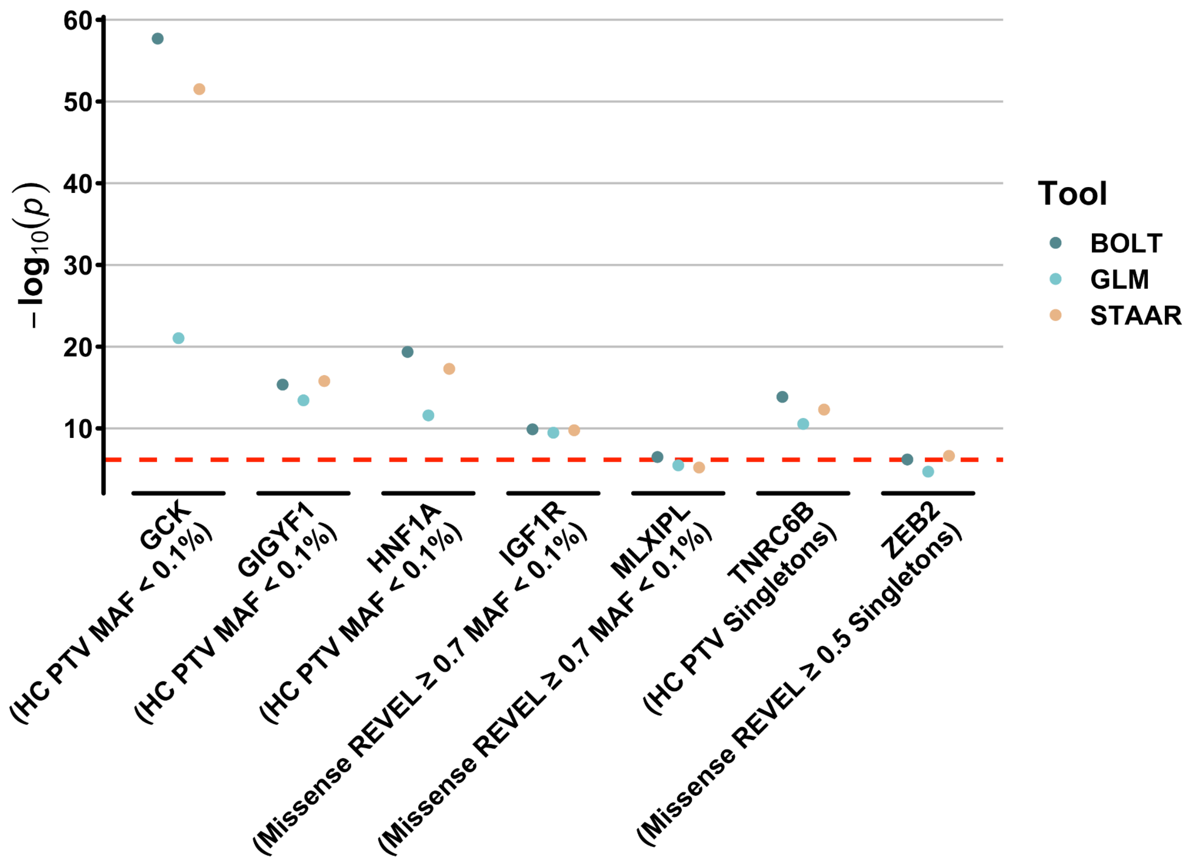

Supplementary Figure S2. Exome-wide association results for additional methods, related to Figure 1.

Displayed are association results for both our primary approach (BOLT) as well as two other orthogonal approaches (generalised linear models (GLM) and STAAR). $-\log_{10}$(p values) for each tool are plotted only for all genes identified as exome-wide significant with BOLT.

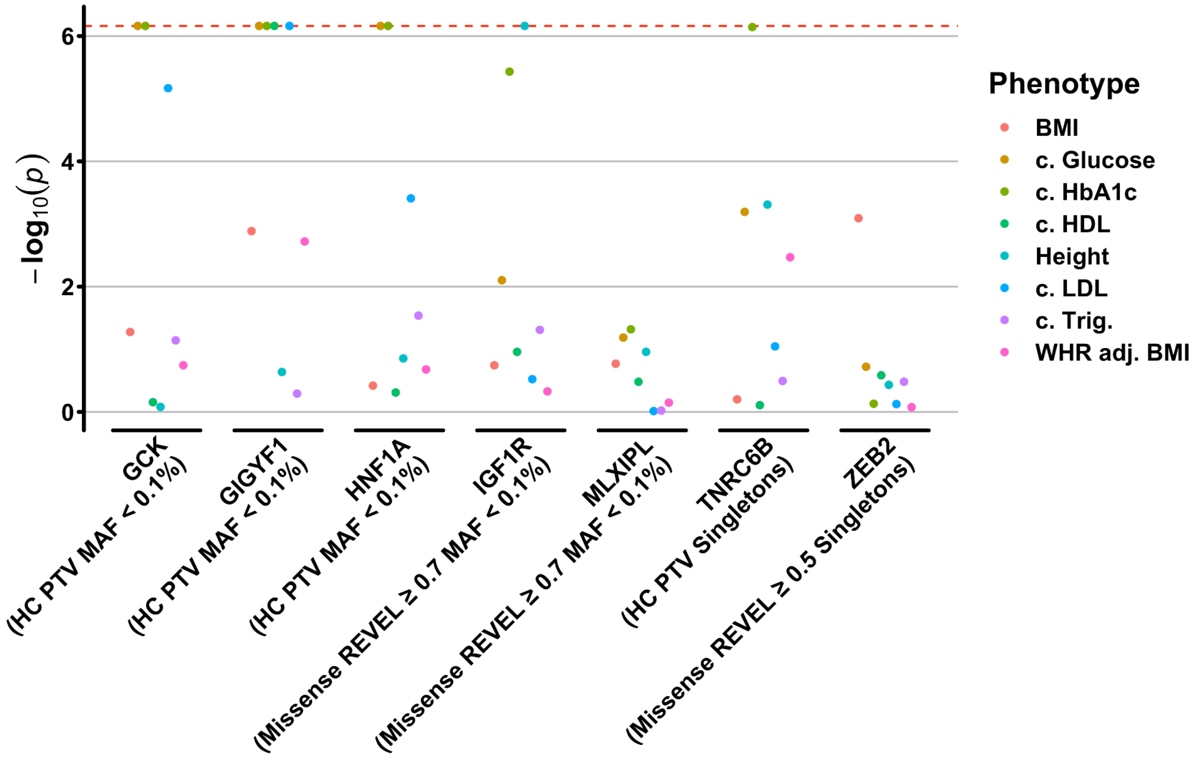

Supplementary Figure S3. Additional phenotypes associated with T2D or the GH-IGF1 pathway, related to Figure 1.

Shown are associations between rare variant burden and eight additional phenotypes with known associations to type 2 diabetes or the GH-IGF1 signalling pathway. Plotted are -$\log_{10}$ p. values for each trait derived from the mask-MAF cutoff most significantly associated with T2D (Main Text Figure 1; Supplementary Table 1). -$\log_{10}$ p. values are capped at exome wide significance (-$\log_{10}$ p = 6.16) to enable comparison of nominally associated signals. ‘c.’, ‘trig.’, and ‘adj.’ in the figure legend stand for ‘circulating’, ‘triglycerides’, and ‘adjusted’, respectively.

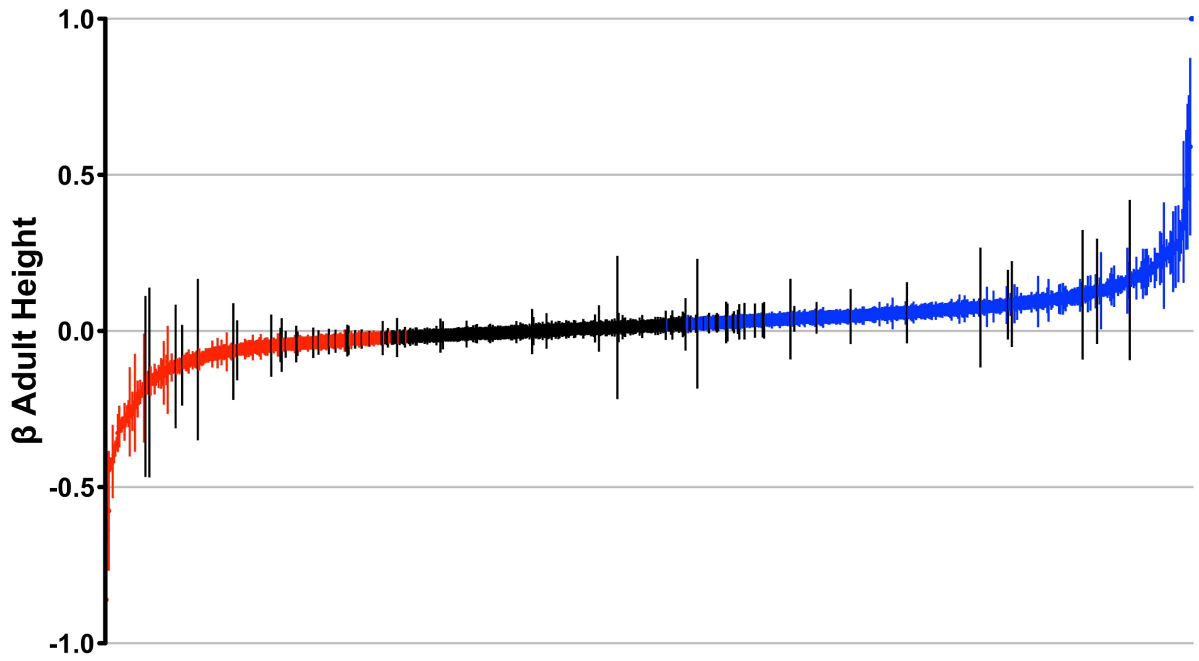

Supplementary Figure S4. Heterogeneity with height of SNPs associated with IGF-1, related to STAR Methods.
Shown are Mendelian randomisation results for 784 independent genetic signals with a known association with circulating IGF-1 levels. SNPs are ordered on the x-axis by their β value for adult height. SNPs in red have a significantly negative association with adult height while SNPs in blue have a significantly positive association with adult height (y-axis). Note that the y-axis has been capped at -1 and +1; two SNPs above this cap in the upper-right hand corner have β's of 1.6 [1.3-1.8] and 1.9 [1.6-2.2]. Error bars represent the 95% confidence interval.
