# Supplementary material for: Damaging missense variants in IGF1R implicate a role for IGF-1 resistance in the etiology of type 2 diabetes
Source: Cell Genom. 2022 Nov 7;2(12):100208. doi: 10.1016/j.xgen.2022.100208 (PMC9750938; doi:10.1016/j.xgen.2022.100208)
Supplement: Document S3. Article plus supplemental information [file mmc8.pdf]

Cell Genomics

Article

Damaging missense variants in *IGF1R* implicate a role for IGF-1 resistance in the etiology of type 2 diabetes

Graphical abstract

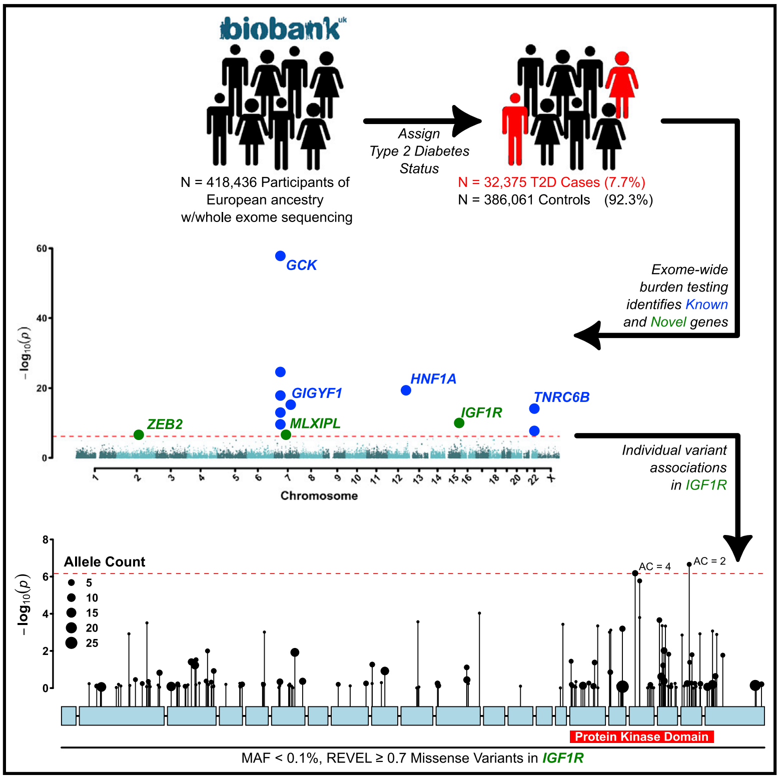

Highlights

- Large-scale T2D exome sequence association study conducted in UK Biobank
- Confirmed T2D associations with rare variant burden in *GCK*, *GIGYF1*, *HNF1A*, and *TNRC6B*
- Identified novel associations in *IGF1R*, *ZEB2*, and *MLXIPL*
- Findings support a role for IGF-1 resistance in T2D etiology

Authors

Eugene J. Gardner, Katherine A. Kentistou, Stasa Stankovic, ..., Stephen O'Rahilly, Ken K. Ong, John R.B. Perry

Correspondence

eugene.gardner@mrc-epid.cam.ac.uk (E.J.G.), john.perry@mrc-epid.cam.ac.uk (J.R.B.P.)

In brief

Gardner et al. queried the genomes of over 400,000 individuals and identified novel genes associated with type 2 diabetes risk. The biological function of these genes highlights potentially new therapeutic avenues for treatment of type 2 diabetes.

Gardner et al., 2022, Cell Genomics *2*, 100208
December 14, 2022 © 2022 The Authors.
https://doi.org/10.1016/j.xgen.2022.100208

CellPress

Cell Genomics

CellPress OPEN ACCESS

Article

Damaging missense variants in *IGF1R* implicate a role for IGF-1 resistance in the etiology of type 2 diabetes

Eugene J. Gardner,[1,7,*] Katherine A. Kentistou,[1] Stasa Stankovic,[1] Samuel Lockhart,[2] Eleanor Wheeler,[1] Felix R. Day,[1] Nicola D. Kerrison,[1] Nicholas J. Wareham,[1] Claudia Langenberg,[1,4] Stephen O'Rahilly,[2,3,8] Ken K. Ong,[1,5,8] and John R.B. Perry[1,6,8,9,*]
[1]MRC Epidemiology Unit, Wellcome-MRC Institute of Metabolic Science, University of Cambridge, Cambridge, UK
[2]MRC Metabolic Diseases Unit, Wellcome-MRC Institute of Metabolic Science, University of Cambridge, Cambridge, UK
[3]NIHR Cambridge Biomedical Research Centre, Cambridge, UK
[4]Computational Medicine, Berlin Institute of Health at Charité-Universitätsmedizin Berlin, Berlin, Germany
[5]Department of Paediatrics, University of Cambridge, Cambridge, UK
[6]Metabolic Research Laboratory, Wellcome-MRC Institute of Metabolic Science, University of Cambridge, Cambridge, UK
[7]Present address: Adrestia Therapeutics, Moneta Building 280, Babraham Research Campus, Cambridge CB22 3AT, UK
[8]Senior author
[9]Lead contact
*Correspondence: eugene.gardner@mrc-epid.cam.ac.uk (E.J.G.), john.perry@mrc-epid.cam.ac.uk (J.R.B.P.)
https://doi.org/10.1016/j.xgen.2022.100208

SUMMARY

Type 2 diabetes (T2D) is a heritable metabolic disorder. While population studies have identified hundreds of common genetic variants associated with T2D, the role of rare (frequency < 0.1%) protein-coding variation is less clear. We performed exome sequence analysis in 418,436 (n = 32,374 T2D cases) individuals in the UK Biobank. We identified previously reported genes (*GCK*, *GIGYF1*, *HNF1A*) in addition to missense variants in *ZEB2* (n = 31 carriers; odds ratio [OR] = 5.5 [95% confidence interval = 2.5–12.0]; $p = 6.4 \times 10^{-7}$), *MLXIPL* (n = 245; OR = 2.3 [1.6–3.2]; $p = 3.2 \times 10^{-7}$), and *IGF1R* (n = 394; OR = 2.4 [1.8–3.2]; $p = 1.3 \times 10^{-10}$). Carriers of damaging missense variants within *IGF1R* were also shorter (−2.2 cm [−1.8 to –2.7]; $p = 1.2 \times 10^{-19}$) and had higher circulating insulin-like growth factor-1 (IGF-1) protein levels (2.3 nmol/L [1.7–2.9]; $p = 2.8 \times 10^{-14}$), indicating relative IGF-1 resistance. A likely causal role of IGF-1 resistance was supported by Mendelian randomization analyses using common variants. These results increase understanding of the genetic architecture of T2D and highlight the growth hormone/IGF-1 axis as a potential therapeutic target.

INTRODUCTION

Type 2 diabetes (T2D) is a complex disease characterized by insulin resistance and β-cell dysfunction. An estimated 630 million adults are expected to have T2D by 2045,[1] making it one of the fastest growing global health challenges of the 21st century. Genome-wide association studies (GWASs) have successfully identified more than 500 genomic loci to be associated with T2D,[2] although the majority of these are driven by common variants with small individual effects on T2D risk.

Over 90% of GWAS loci lie in non-coding regions of the genome,[3] presenting a major hurdle for the identification of the underlying causal genes and the translation of these findings into mechanistic insight. In contrast, analysis of rare protein-coding variation captured by DNA sequencing has the potential to more directly implicate individual genes and biological mechanisms. The UK Biobank (UKBB)[4] study recently made exome sequencing (ES) data available for 454,787 UKBB participants.[5] This offers an unprecedented opportunity to explore the contribution of rare coding variations to the risk of T2D with much greater power than previously possible.[6–8] Initial exome-wide association analyses of these data have identified gene-based associations with increased risk of T2D for *GCK*, *HNF1A*, *HNF4A*, *GIGYF1*, *CCAR2*, *TNRC6B*, and *PAM* and protective effects for variants in *FAM234A* and *MAP3K15*.[5,9–14]

In this study, we combined multiple sources of health record data to identify additional T2D cases and used an extended range of variant classes and allele frequency cutoffs in order to directly implicate novel genes in the etiology of T2D. Our results highlight a number of previously missed associations and support a role for insulin-like growth factor 1 (IGF-1) resistance in the pathogenesis of T2D.

RESULTS

Exome-wide burden testing in the UKBB

To identify genes associated with T2D risk, we performed an exome-wide association study (ExWAS) using ES data derived

© 2022 The Authors.
This is an open access article under the CC BY license (http://creativecommons.org/licenses/by/4.0/).

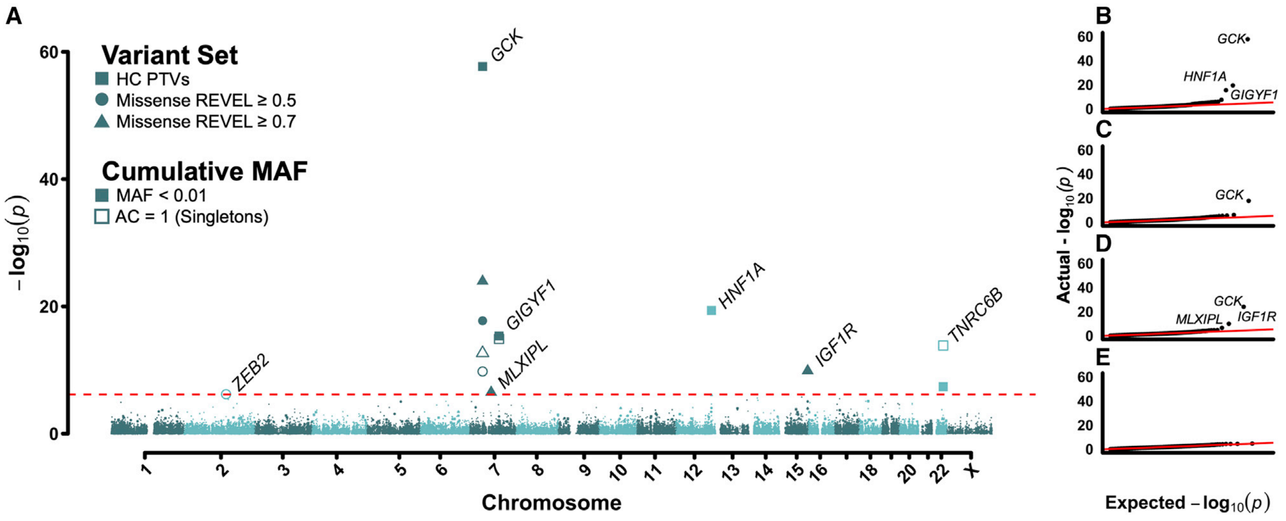

Figure 1. Exome-wide association results for T2D

(A) Manhattan plot displaying results of gene burden tests for T2D risk (n = 418,436 participants). Genes passing exome-wide significance based on per-gene burden tests as implemented in BOLT-LMM[17] ($p < 6.9 \times 10^{-7}$) are labeled. Point shape indicates variant class tested.

(B–E) QQ plots for (B) high-confidence PTVs, (C) REVEL ≥0.5 missense variants, (D) REVEL ≥0.7 missense variants, and (E) synonymous variants (negative control).

from 418,436 European genetic ancestry UKBB participants.[5] As our primary outcome, we identified 32,374 (7.7%) participants with likely incident or prevalent T2D using phenotype curation that integrated multiple data sources, including hospital episode statistics, self-reported conditions, death records, and use of T2D medication (see STAR Methods).

Individual gene burden tests were performed by collapsing genetic variants across 18,691 protein-coding genes in the human genome. We tested four functional categories across two population prevalences (minor allele frequency <0.1% and singletons), including high-confidence protein-truncating variants (PTVs), missense variants stratified by two REVEL score thresholds,[15] and synonymous variants as a negative control (Figure 1; STAR Methods). We identified 13 gene-functional annotation pairs with 30 or more rare allele carriers, representing 7 non-redundant genes, associated with T2D at exome-wide statistical significance ($p < 6.9 \times 10^{-7}$; Table S1; STAR Methods). Our results are statistically well calibrated, as indicated by both low exome-wide inflation scores (e.g., PTV $\lambda = 1.047$) and the absence of significant associations with synonymous variant burden (Figures 1B–1E and S1). To ensure our results were not biased by our approach, we implemented burden tests using both STAAR[16] and a logistic model and arrived at substantially similar conclusions (Figure S2; Table S1; STAR Methods).

We confirmed the T2D associations at all three genes identified by three previous studies of T2D risk that incorporated European genetic ancestry individuals from the UKBB study:[11,12,14] *GCK* (n = 35 carriers; odds ratio [OR] = 58.5 [95% confidence interval (CI) = 25.5–134.5]; $p = 2.0 \times 10^{-58}$), *HNF1A* (n = 33; OR = 12.7 [6.2–25.8]; $p = 4.4 \times 10^{-20}$), and *GIGYF1* (n = 133; OR = 4.7 [3.1–7.0]; $p = 4.4 \times 10^{-16}$; Figure 2). As in these previous studies, we similarly found that carriers of PTVs within these genes had substantially increased risk for developing T2D (Figure 1A). In support of other recent studies,[18] we note surprisingly low penetrance rates for *GCK* (77% of PTV carriers with T2D) and *HNF1A* (48%) for effects that are clinically considered to be highly/completely penetrant for monogenic forms of maturity-onset diabetes of the young (MODY; Table S1). Future studies will be needed to better estimate the true penetrance, which will lie somewhere between the likely underestimates obtained from healthy population studies such as the UKBB[19] and the inflated estimates obtained from clinical genetics referrals.

We also confirmed the T2D association at *TNRC6B* (n = 35; OR = 10.5 [5.3–21.0]; $p = 1.4 \times 10^{-14}$), which was previously reported as "potentially spuriously associated" with T2D risk;[11] several additional lines of evidence provide confidence in this association. Firstly, our result is not attributable to a single variant of large effect as evidenced by the strength of association with singleton variants (Figure 1A). Secondly, aside from a single individual carrying two balanced deletions, inspection of the underlying ES reads did not reveal a markedly increased error rate in variant calling or genotyping in *TNRC6B* as was suggested by Deaton et al.[11] Thirdly, the association persisted after excluding 14 individuals who carry a singleton PTV in a potentially non-constitutive exon as measured by the proportion expressed across transcripts score ($p = 3.6 \times 10^{-7}$).[20] Finally, we also found that *TNRC6B* PTV carriers had elevated HbA1c levels when considering both T2D cases (4.1 mmol/mol [2.5–5.7]; $p = 7.2 \times 10^{-7}$; Figure S3) and controls (1.6 mmol/mol [0.2–2.1]; $p = 1.8 \times 10^{-2}$), consistent with the elevated long-term blood glucose levels observed in individuals with T2D.

We also identified three additional genes that, when disrupted by rare genetic variation (minor allele frequency <0.1% or singletons), are associated with increased T2D risk: *IGF1R* (n = 394; OR = 2.4 [1.8–3.2]; $p = 1.3 \times 10^{-10}$), *MLXIPL*

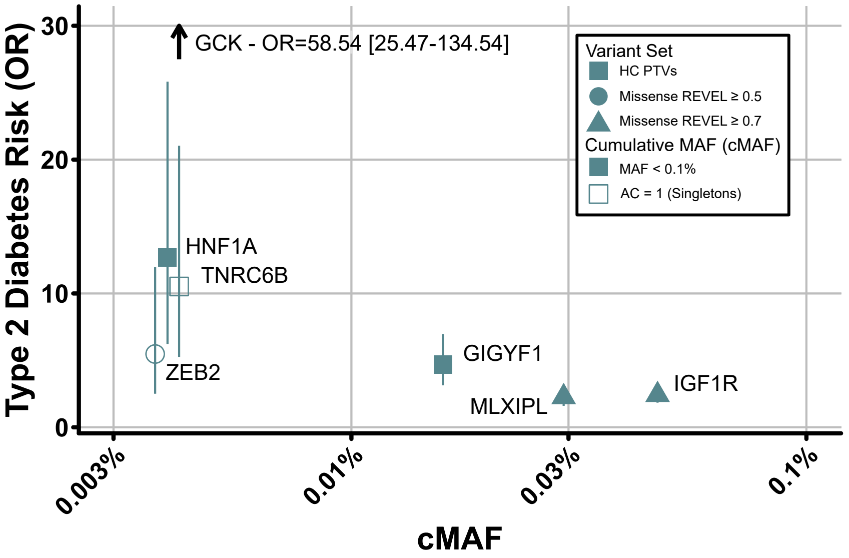

Figure 2. Relationship between cumulative minor allele frequency and odds ratio for T2D

Plotted is T2D risk as quantified by odds ratio versus cumulative minor allele frequencies (cMAFs) for genes significantly associated with T2D risk (n = 418,436 participants). For each gene, only the most significantly associated variant mask is shown. Error bars indicate 95% confidence intervals.

(n = 245; OR = 2.3 [1.6–3.2]; p = 3.2 × 10^{-7}), and *ZEB2* (n = 31; OR = 5.5 [2.5–12.0]; p = 6.4 × 10^{-7}; Figure 1). Unlike previously reported genes outlined above, damaging missense variants, but not PTVs, in these genes were associated with T2D risk (Figure 2). Indeed, in these genes, T2D associations were apparent only with missense variants with high REVEL scores (≥0.7) or those variants considered to be the most damaging as per current (2020) Association for Clinical Genomic Science guidelines.

Specifically, and as expected, we found that carriers of PTVs within *GCK*, *GIGYF1*, and *HNF1A* all had significantly elevated circulating glucose and HbA1c levels. Among novel genes, *IGF1R* missense carriers had nominally higher HbA1c levels (1.1 mmol/mol [0.6–1.6]; p = 3.7 × 10^{-6}).

Exploring common variant associations at highlighted genes

We next attempted to cross-validate the rare-variant associations for all seven exome-wide significant genes by identifying proximal common variants (±50 kb of a gene's coding sequence) previously reported to be associated with related glycemic or metabolic phenotypes (STAR Methods; Table S2). Four genes fell within glycemic trait-associated loci, and all seven overlapped known metabolic trait associations. For several of these common variant-phenotype combinations, we also identified an association with rare variant burden (Figure S3). Additionally, three of the four novel genes we report here were identified in the most recent publicly available T2D GWAS[2] as being either the closest or most likely causal gene for a common variant genome-wide significant signal: *IGF1R*, *TNRC6B*, and *ZEB2* (Table S2).

Notably, common non-coding variants at the *IGF1R* locus have been previously reported for T2D and fasting glucose.[2,21] The lead fasting glucose-associated SNP (rs6598541-A; p = 4 × 10^{-12}) was associated with 0.0114 mmol/L (0.0097–0.0131) higher levels of glucose, while the lead T2D SNP (rs59646751-T; p = 4 × 10^{-9}) increases risk of T2D by an odds ratio of 1.024 (1.020–1.028). Both SNPs are intronic in *IGF1R*, in moderate linkage disequilibrium in European populations (R^2 = 75.5%),[22] and are expression quantitative trait loci (eQTLs) for *IGF1R*.[23] Furthermore, for both SNPs, the *IGF1R* expression-lowering alleles are associated with higher levels of circulating IGF-1 (p = 7 × 10^{-7} and 9 × 10^{-7}, respectively; Figure 3)[24] and with higher T2D risk and fasting glucose, although colocalization analyses could not confirm that these effects were driven by the same signals (PP3 = 1, PP4 = 2 × 10^{-6}; Figure 3), likely due to the presence of multiple independent signals. This result, in light of our rare variant association, highlights the limitations of reliance on common variant approaches.

Interrogating *IGF1R* and risk for T2D

To understand how rare damaging missense variants in *IGF1R* lead to increased risk of T2D, we performed burden tests for circulating IGF-1 levels and anthropometric traits. We found that carriers of damaging missense variants in *IGF1R* had higher circulating IGF-1 levels (2.1 nmol/L [1.5–2.6]; p = 1.9 × 10^{-14}) but shorter adult stature (−2.2 cm [−1.8 to −2.7]; p = 1.2 × 10^{-19}) and lower relative height at age 10 (p = 1.1 × 10^{-7}). These findings indicate that carriers of rare damaging missense variants in *IGF1R* that increase risk of T2D have relative IGF-1 resistance.

To explore how damaging missense variants disrupt *IGF1R* function, we next categorized variants by protein domain. Carriers of qualifying variants within the *IGF1R* protein kinase (residues 999–1274)[25] had a higher risk for T2D (n = 179; OR = 3.4 [2.3–4.9]; p = 1.9 × 10^{-10}) than those with qualifying variants outside this domain (n = 215; OR = 1.7 [1.2–2.6]; p = 8.2 × 10^{-3}). This difference is not statistically significant in the current sample (heterogeneity p = 0.40); however, we speculate that dysfunction within the protein kinase domain might decrease downstream signal transduction resulting in IGF-1 resistance. This may also explain why, despite the relatively large number of *IGF1R* (n = 64) PTV carriers in the UKBB, we did not find that *IGF1R* PTV carriers had increased T2D risk. When bound by IGF-1 and to induce downstream signal transduction, *IGF1R* functions as a homo- or heterodimer (i.e., with *INSR* as a hybrid receptor).[26] As half of a missense carrier's *IGF1R* molecules will contain errors in the protein kinase domain, dimerization will incorporate at least one defective molecule 75% of the time and therefore lead to reduced downstream signal transduction. In the case of PTV carriers, since one copy is likely missing due to nonsense-mediated decay, dimerization will always

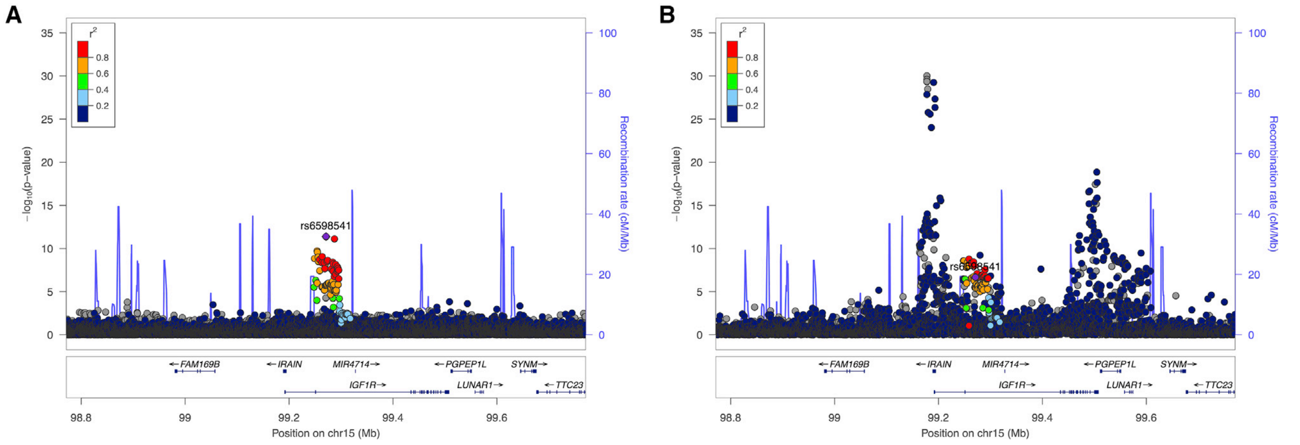

Figure 3. Common variant associations at the *IGF1R* locus
Association pattern between SNPs at the *IGF1R* locus and (A) fasting glucose levels and (B) IGF-1 levels.

incorporate two functional copies. Therefore, the association of damaging *IGF1R* missense variants with T2D may be due to a dominant-negative effect rather than decreased protein abundance; however, additional functional studies are ultimately required to confirm the mechanism underlying these variants.

To explore whether rare variants in other components of the growth hormone (GH)-IGF1 hormone pathway might influence T2D risk, we next identified a further nine genes in the GH-IGF1 pathway that showed gene-burden associations with circulating IGF-1 levels in any of our burden tests (Table S3), including seven genes with known roles in regulating GH secretion or GH signaling and three genes with known roles in IGF-1 bioavailability. We tested their associations with childhood and adult height to indicate the functional relevance of rare variations in these genes. None of the seven GH-related genes showed any association with T2D. Rare damaging variants in *IGFALS*, which encodes a component of the IGF-1 ternary complex, lowered circulating IGF-1 and were nominally associated with shorter childhood height (indicative of lower IGF-1 bioactivity) and higher risk of T2D. Rare damaging variants in *IGFBP3* (the major IGF binding protein), which lowered circulating IGF-1 levels, were nominally associated with taller childhood height (indicative of higher IGF-1 bioactivity) and lower risk of T2D. Hence, damaging rare variants that disrupt IGF-1 bioactivity, but not those that primarily alter GH secretion or signaling, appear to increase T2D risk.

Causality of IGF-1 levels with T2D risk

A previous phenotypic observational study described a protective association between baseline circulating IGF-1 protein levels and incident T2D.[27] However, subsequent similar studies found no such association,[28,29] and conversely, a previous study that modeled common genetic variants in a Mendelian randomization framework inferred an adverse causal effect of higher circulating IGF-1 levels on T2D.[30]

To explore this apparent inconsistency, we examined the likely causal role of IGF-1 on T2D by modeling 784 independent common genetic signals for circulating IGF-1 levels identified in 428,525 White European UKBB individuals[24] and summary statistics from the largest reported GWAS meta-analysis of T2D.[31] We confirmed the previously reported[30] association between genetically predicted higher IGF-1 levels and higher risk of T2D in inverse-variance weighted (IVW; OR = 1.105 per SD [95% CI 1.039–1.170]; $p = 2.9 \times 10^{-3}$) and sensitivity models (Table S4). However, we noted substantial heterogeneity in the relationships between individual IGF-1 signals and T2D (I-square = 85.7%) as well as in their associations with adult height (IVW beta = 0.142; $p = 8.9 \times 10^{-9}$; I-square = 97.7%). Among the common genetic instruments for higher circulating IGF-1 levels, individual variants at the *IGF1* locus (rs11111274) and the *IGF1R* locus (rs1815009) show directionally opposite effects on childhood height and T2D (taller height and lower T2D risk for *IGF1*; shorter height and higher T2D risk for *IGF1R*; Figure S4). Hence, reported common variant instruments for higher IGF-1 levels comprise a mixture of functionally opposing signals, i.e., higher levels of bioactive IGF-1 or higher IGF-1 resistance.

DISCUSSION

Here, we present the results of an ExWAS to assess the contribution of rare variant burden to T2D risk (Figure 1). We identified three genes previously reported by a recent analysis of the UKBB (*GCK*, *HNF1A*, and *GIGYF1*),[14] provided stronger evidence for a previously nominally associated gene (*TNRC6B*),[11] and identified three new genes (*ZEB2*, *MLXIPL*, and *IGF1R*) where rare variants increase susceptibility to T2D (Figure 2). Using publicly available data, we showed that common variation nearby these genes is associated with a wide range of glycemic and metabolic traits (Figure 3; Table S2),[2,21] providing further support for these rare variant associations. We further interrogated rare and common variant associations to show that disruption of *IGF1R* due to damaging missense variants in the cytoplasmic protein kinase domain leads to IGF-1 resistance and higher T2D risk. Overall, our results implicate a wider protective effect of IGF-1 bioactivity on susceptibility to T2D.

While our results are complementary to previous ExWAS,[11,14] we clarified evidence linking *TNRC6B* to T2D and identified three

additional genes missed by previous analyses of the UKBB. A key advantage of our approach was to carefully curate multiple data sources to identify and validate T2D cases. Furthermore, we used a different genetic analytical approach from those previous studies. Nag et al.[14] limited their burden testing to either PTVs, the findings of which we replicate here, or to missense variants with comparatively low deleteriousness scores (REVEL >0.25 or missense tolerance ratio intragenic percentiles ≤50%). In this study, we have shown the benefit of considering missense variants computationally predicted to be severely damaging (REVEL ≥0.5 and 0.7).[15] While such variants are much rarer in the population—only ~8% of missense variants in UKBB have REVEL scores ≥0.7—they are much more likely to disrupt protein function and thus increase risk for disease. These conclusions are similar to those shown previously for anthropometric traits,[10] which have shown a relationship between PTVs in *IGF1R* and several growth measures, but not for damaging missense variants.

A key finding of our work is the association between *IGF1R* and T2D risk. Loss-of-function mutations in *IGF1R* have been reported in children presenting with intrauterine growth restriction, short stature, and elevated IGF-1 levels.[32–34] Our findings of rare damaging variants at *IGF1R*, and also at *IGFALS* and *IGFBP3*, indicate that reduced IGF-1 bioactivity and signaling increases risk for T2D. There are several plausible mechanisms to link *IGF1R* to T2D. *IGF1R*, responding to both systemic and locally generated IGF-1, plays a role in the development of several tissues central to the control of glucose metabolism including pancreatic islets, adipose tissue, and skeletal muscle.[35] An alternative explanation involves the complex relationship between GH and IGF-1. GH, produced in a highly controlled and pulsatile manner from the somatotropes of the anterior pituitary, is the major stimulus to the hepatic expression and secretion of IGF-1, the major source of this circulating hormone. GH also has metabolic effects that are independent of IGF-1, largely exerted by its powerful lipolytic effects in adipose tissue,[36–41] which, if uncontrolled, can lead to the accumulation of ectopic lipid in non-adipose tissue, resulting in insulin resistance. This is elegantly demonstrated by studies in mice in which IGF-1 is selectively deleted in the liver.[42,43] These mice show a striking increase in circulating GH levels accompanied by marked insulin resistance, which is entirely abrogated by the blockade of GH signaling. This model can explain the insulin resistance and frequent T2D seen in conditions such as acromegaly, where GH levels are persistently raised due to a functional somatotrope tumor,[44] and the striking protection from T2D seen in patients with Laron dwarfism, whose markedly reduced circulating IGF-1 levels are due to biallelic loss-of-function (LoF) mutations in the GH receptor.[45] LoF mutations in *IGF1R* are likely to result in compensatory increases in GH secretion and, consequently, the higher levels of circulating IGF-1 that we observed in the carriers of such mutations. While this may partially compensate for impairment in *IGF1R* function, the lipolytic effects of GH are likely to have a deleterious effect on systemic glucose metabolism. Of note in this regard, a single human proband with a homozygous LoF mutation in *IGF1* had elevated circulating GH and severe insulin resistance.[46,47] Therapy with exogenous IGF-1 resulted in suppression of GH and a dose-dependent improvement in insulin sensitivity.[47] Accordingly, genetic variants that primarily reduce GH secretion and signaling would lead to reduced IGF-1 bioactivity but without the consequent effects of elevated GH on fatty acid metabolism and insulin resistance and, hence, no alteration in T2D risk. We propose that currently available drugs that reduce GH secretion or block its action may have metabolic benefits in patients with T2D and damaging missense variants in the protein kinase domain of *IGF1R*.

Our findings also demonstrate the challenge of interpreting Mendelian randomization results of circulating biomarkers. Elevated biomarker levels may reflect higher levels of secretion and biomarker activity or can be increased by mechanisms that reduce biomarker bioavailability or sensitivity. Hence, genetic instruments for higher biomarker levels may comprise a mixture of markers for higher or lower biomarker activity. To distinguish these actions, we suggest that individual common variants are first tested for association with some indicator of biomarker activity (i.e., childhood height as an indicator of IGF-1 activity).

Our rare variant analysis also implicates *MLXIPL* as a T2D susceptibility gene for the first time. *MLXIPL* encodes the carbohydrate response element-binding protein (CHREBP), a transcription factor that acts in concert with its obligate binding partner MLX to regulate the cellular response to carbohydrate[48–50] and is highly expressed in liver, fat, and muscle. Global or tissue-specific ablation of *MLXIPL* in mice impairs insulin sensitivity.[51–54] Common variants at the *MLXIPL* locus associate with *SHBG*, a biomarker of insulin sensitivity[55] and with serum triglycerides.[56,57] Notably, *MLXIPL* is one of the 26–28 genes deleted in Williams syndrome, the result of a deletion of contiguous genes on chromosome 7q11.23. Patients with this syndrome are characterized by marked insulin resistance and an increased risk of diabetes.[58] It seems likely that haploinsufficiency for *MXLIPL* contributes to the metabolic disturbances characteristic of Williams syndrome.

We acknowledge several limitations of our study. Independent replication was restricted by the limited availability of similar large whole-exome sequencing (WES) studies, although common variant associations at *IGF1R*, *TNRC6B*, and *ZEB2* provide some confirmation that these genes contribute to T2D etiology. We relied on *in silico* predictions of the functional consequences of rare variants, and future work is required to experimentally characterize their impacts on protein function and specifically to test the hypothesized dominant-negative effect of missense variants in *IGF1R*. Finally, it is recognized that the UKBB sample is affected by "healthy volunteer bias,"[59] and this may potentially attenuate true disease associations.

Overall, our findings suggest that deeper interrogation of multiple variant types when performing ExWAS can and will lead to the discovery of additional genes associated with a wide range of human diseases.

STAR★METHODS

Detailed methods are provided in the online version of this paper and include the following:

- KEY RESOURCES TABLE

- RESOURCE AVAILABILITY
  - Lead contact
  - Materials availability
  - Data and code availability
- METHOD DETAILS
  - UK biobank data processing and quality control
  - Exome-wide association analyses in the UK biobank
  - Common variant GWAS lookups
  - Mendelian randomisation using IGF-1 levels

SUPPLEMENTAL INFORMATION

Supplemental information can be found online at https://doi.org/10.1016/j.xgen.2022.100208.

ACKNOWLEDGMENTS

This work was funded by the Medical Research Council (unit programs: MC_UU_12015/2, MC_UU_00006/2, MC_UU_12015/1, and MC_UU_00006/1). This research was supported by the NIHR Cambridge Biomedical Research Centre (BRC-1215-20014). S.L. is supported by a Wellcome Trust Clinical PhD Fellowship (225479/Z/22/Z). S.O. is supported by a Wellcome Investigator Award (214274/Z/19/Z). For the purpose of open access, the author has applied a Creative Commons Attribution (CC BY) license to any author accepted manuscript version arising. This research was conducted using the UKBB Resource under application 9905.

AUTHOR CONTRIBUTIONS

E.J.G. performed WES quality control, variant annotation, and rare variant burden tests, performed statistical modelling, and analyzed primary data. K.A.A. assessed common variants associated with T2D and associated phenotypes. S.S. and K.K.O. performed Mendelian randomization experiments. S.L. and S.O. provided clinical insight and scientific direction regarding phenotypes associated with carriers of *IGF1R* and *MLXIPL* missense variants. E.W., N.D.K., N.J.W., and C.L. generated the T2D phenotype used in this study. E.J.G., F.R.D., and K.A.A. queried and curated additional UKBB participant phenotypic data used in this study. E.J.G., S.O., K.K.O., and J.R.B.P. designed experiments, oversaw the study, and wrote the manuscript.

DECLARATION OF INTERESTS

E.J.G. and J.R.B.P. are employees of and hold shares in Adrestia Therapeutics.

Received: April 14, 2022
Revised: August 12, 2022
Accepted: October 7, 2022
Published: November 7, 2022

REFERENCES

1. International Diabetes Federation (2021). IDF Diabetes Atlas, Ninth edition (International Diabetes Federation).
2. Vujkovic, M., Keaton, J.M., Lynch, J.A., Miller, D.R., Zhou, J., Tcheandjieu, C., Huffman, J.E., Assimes, T.L., Lorenz, K., Zhu, X., et al. (2020). Discovery of 318 new risk loci for type 2 diabetes and related vascular outcomes among 1.4 million participants in a multi-ancestry meta-analysis. Nat. Genet. *52*, 680–691.
3. Loos, R.J.F. (2020). 15 years of genome-wide association studies and no signs of slowing down. Nat. Commun. *11*, 5900.
4. Szustakowski, J.D., Balasubramanian, S., Kvikstad, E., Khalid, S., Bronson, P.G., Sasson, A., Wong, E., Liu, D., Wade Davis, J., Haefliger, C., et al. (2021). Advancing human genetics research and drug discovery through exome sequencing of the UK Biobank. Nat. Genet. *53*, 942–948.
5. Backman, J.D., Li, A.H., Marcketta, A., Sun, D., Mbatchou, J., Kessler, M.D., Benner, C., Liu, D., Locke, A.E., Balasubramanian, S., et al. (2021). Exome sequencing and analysis of 454, 787 UK Biobank participants. Nature *599*, 628–634.
6. Fuchsberger, C., Flannick, J., Teslovich, T.M., Mahajan, A., Agarwala, V., Gaulton, K.J., Ma, C., Fontanillas, P., Moutsianas, L., McCarthy, D.J., et al. (2016). The genetic architecture of type 2 diabetes. Nature *536*, 41–47.
7. Flannick, J., Mercader, J.M., Fuchsberger, C., Udler, M.S., Mahajan, A., Wessel, J., Teslovich, T.M., Caulkins, L., Koesterer, R., Barajas-Olmos, F., et al. (2019). Exome sequencing of 20, 791 cases of type 2 diabetes and 24, 440 controls. Nature *570*, 71–76.
8. Langenberg, C., and Lotta, L.A. (2018). Genomic insights into the causes of type 2 diabetes. Lancet *391*, 2463–2474.
9. Curtis, D. (2022). Analysis of rare coding variants in 200, 000 exome-sequenced subjects reveals novel genetic risk factors for type 2 diabetes. Diabetes. Metab. Res. Rev. *38*, e3482.
10. Wang, Q., Dhindsa, R.S., Carss, K., Harper, A.R., Nag, A., Tachmazidou, I., Vitsios, D., Deevi, S.V.V., Mackay, A., Muthas, D., et al. (2021). Rare variant contribution to human disease in 281, 104 UK Biobank exomes. Nature *597*, 527–532.
11. Deaton, A.M., Parker, M.M., Ward, L.D., Flynn-Carroll, A.O., BonDurant, L., Hinkle, G., Akbari, P., Lotta, L.A., Regeneron Genetics Center, DiscovEHR Collaboration, et al. (2021). Gene-level analysis of rare variants in 379, 066 whole exome sequences identifies an association of GIGYF1 loss of function with type 2 diabetes. Sci. Rep. *11*, 21565.
12. Zhao, Y., Stankovic, S., Koprulu, M., Wheeler, E., Day, F.R., Lango Allen, H., Kerrison, N.D., Pietzner, M., Loh, P.-R., Wareham, N.J., et al. (2021). GIGYF1 loss of function is associated with clonal mosaicism and adverse metabolic health. Nat. Commun. *12*, 4178.
13. Jurgens, S.J., Choi, S.H., Morrill, V.N., Chaffin, M., Pirruccello, J.P., Halford, J.L., Weng, L.-C., Nauffal, V., Roselli, C., Hall, A.W., et al. (2022). Analysis of rare genetic variation underlying cardiometabolic diseases and traits among 200, 000 individuals in the UK Biobank. Nat. Genet. *54*, 240–250. https://doi.org/10.1038/s41588-021-01011-w.
14. Nag, A., Dhindsa, R.S., Harper, A.R., Vitsios, D., Ahnmark, A., Bilican, B., Madeyski-Bengtson, K., Zarrouki, B., Wang, Q., Smith, K., et al. (2021). Human genetic evidence supports MAP3K15 inhibition as a therapeutic strategy for diabetes. Preprint at medRxiv. https://doi.org/10.1101/2021.11.14.21266328.
15. Ioannidis, N.M., Rothstein, J.H., Pejaver, V., Middha, S., McDonnell, S.K., Baheti, S., Musolf, A., Li, Q., Holzinger, E., Karyadi, D., et al. (2016). REVEL: an ensemble method for predicting the pathogenicity of rare missense variants. Am. J. Hum. Genet. *99*, 877–885.
16. Li, X., Li, Z., Zhou, H., Gaynor, S.M., Liu, Y., Chen, H., Sun, R., Dey, R., Arnett, D.K., Aslibekyan, S., et al. (2020). Dynamic incorporation of multiple in silico functional annotations empowers rare variant association analysis of large whole-genome sequencing studies at scale. Nat. Genet. *52*, 969–983.
17. Loh, P.-R., Tucker, G., Bulik-Sullivan, B.K., Vilhjálmsson, B.J., Finucane, H.K., Salem, R.M., Chasman, D.I., Ridker, P.M., Neale, B.M., Berger, B., et al. (2015). Efficient Bayesian mixed-model analysis increases association power in large cohorts. Nat. Genet. *47*, 284–290.
18. Mirshahi, U.L., Colclough, K., Wright, C.F., Wood, A.R., Beaumont, R.N., Tyrrell, J., et al. (2022). Reduced penetrance of MODY-associated HNF1A/HNF4A variants but not GCK variants in clinically unselected cohorts. Am. J. Hum. Genet. https://doi.org/10.1016/j.ajhg.2022.09.014.
19. Lam, B.Y.H., Williamson, A., Finer, S., Day, F.R., Tadross, J.A., Gonçalves Soares, A., Wade, K., Sweeney, P., Bedenbaugh, M.N., Porter, D.T., et al. (2021). MC3R links nutritional state to childhood growth and the timing of puberty. Nature *599*, 436–441.
20. Cummings, B.B., Karczewski, K.J., Kosmicki, J.A., Seaby, E.G., Watts, N.A., Singer-Berk, M., Mudge, J.M., Karjalainen, J., Satterstrom, F.K.,

CellPress
OPEN ACCESS

O'Donnell-Luria, A.H., et al. (2020). Transcript expression-aware annotation improves rare variant interpretation. Nature *581*, 452–458.

21. Chen, J., Spracklen, C.N., Marenne, G., Varshney, A., Corbin, L.J., Luan, J., Willems, S.M., Wu, Y., Zhang, X., Horikoshi, M., et al. (2021). The trans-ancestral genomic architecture of glycemic traits. Nat. Genet. *53*, 840–860.
22. Machiela, M.J., and Chanock, S.J. (2015). LDlink: a web-based application for exploring population-specific haplotype structure and linking correlated alleles of possible functional variants. Bioinformatics *31*, 3555–3557.
23. GTEx Consortium (2020). The GTEx Consortium atlas of genetic regulatory effects across human tissues. Science *369*, 1318–1330.
24. Stankovic, S., R Day, F., Zhao, Y., Langenberg, C., J Wareham, N., R B Perry, J., K Ong, K., and Ong, K.K. (2021). Elucidating the genetic architecture underlying IGF1 levels and its impact on genomic instability and cancer risk. Wellcome Open Res. *6*, 20. https://doi.org/10.12688/wellcomeopenres.16417.1.
25. Favelyukis, S., Till, J.H., Hubbard, S.R., and Miller, W.T. (2001). Structure and autoregulation of the insulin-like growth factor 1 receptor kinase. Nat. Struct. Biol. *8*, 1058–1063.
26. Li, J., Choi, E., Yu, H., and Bai, X.-C. (2019). Structural basis of the activation of type 1 insulin-like growth factor receptor. Nat. Commun. *10*, 4567.
27. Sandhu, M.S., Heald, A.H., Gibson, J.M., Cruickshank, J.K., Dunger, D.B., and Wareham, N.J. (2002). Circulating concentrations of insulin-like growth factor-I and development of glucose intolerance: a prospective observational study. Lancet *359*, 1740–1745.
28. Lewitt, M.S., Hilding, A., Brismar, K., Efendic, S., Ostenson, C.-G., and Hall, K. (2010). IGF-binding protein 1 and abdominal obesity in the development of type 2 diabetes in women. Eur. J. Endocrinol. *163*, 233–242.
29. Similä, M.E., Kontto, J.P., Virtamo, J., Hätönen, K.A., Valsta, L.M., Sundvall, J., and Männistö, S. (2019). Insulin-like growth factor I, binding proteins -1 and -3, risk of type 2 diabetes and macronutrient intakes in men. Br. J. Nutr. *121*, 938–944.
30. Larsson, S.C., Michaëlsson, K., and Burgess, S. (2020). IGF-1 and cardiometabolic diseases: a Mendelian randomisation study. Diabetologia *63*, 1775–1782.
31. Mahajan, A., Taliun, D., Thurner, M., Robertson, N.R., Torres, J.M., Rayner, N.W., Payne, A.J., Steinthorsdottir, V., Scott, R.A., Grarup, N., et al. (2018). Fine-mapping type 2 diabetes loci to single-variant resolution using high-density imputation and islet-specific epigenome maps. Nat. Genet. *50*, 1505–1513.
32. Abuzzahab, M.J., Schneider, A., Goddard, A., Grigorescu, F., Lautier, C., Keller, E., Kiess, W., Klammt, J., Kratzsch, J., Osgood, D., et al. (2003). IGF-I receptor mutations resulting in intrauterine and postnatal growth retardation. N. Engl. J. Med. *349*, 2211–2222.
33. Fang, P., Cho, Y.H., Derr, M.A., Rosenfeld, R.G., Hwa, V., and Cowell, C.T. (2012). Severe short stature caused by novel compound heterozygous mutations of the insulin-like growth factor 1 receptor (IGF1R). J. Clin. Endocrinol. Metab. *97*, E243–E247.
34. Inagaki, K., Tiulpakov, A., Rubtsov, P., Sverdlova, P., Peterkova, V., Yakar, S., Terekhov, S., and LeRoith, D. (2007). A familial insulin-like growth factor-I receptor mutant leads to short stature: clinical and biochemical characterization. J. Clin. Endocrinol. Metab. *92*, 1542–1548.
35. Rother, K.I., and Accili, D. (2000). Role of insulin receptors and IGF receptors in growth and development. Pediatr. Nephrol. *14*, 558–561.
36. Kopchick, J.J., Berryman, D.E., Puri, V., Lee, K.Y., and Jorgensen, J.O.L. (2020). The effects of growth hormone on adipose tissue: old observations, new mechanisms. Nat. Rev. Endocrinol. *16*, 135–146.
37. Arlien-Søborg, M.C., Dal, J., Madsen, M.A., Høgild, M.L., Hjelholt, A.J., Pedersen, S.B., Møller, N., Jessen, N., and Jørgensen, J.O.L. (2022). Reversible insulin resistance in muscle and fat unrelated to the metabolic syndrome in patients with acromegaly. EBioMedicine *75*, 103763.
38. Nielsen, S., Møller, N., Christiansen, J.S., and Jørgensen, J.O. (2001). Pharmacological antilipolysis restores insulin sensitivity during growth hormone exposure. Diabetes *50*, 2301–2308.
39. Høyer, K.L., Høgild, M.L., List, E.O., Lee, K.Y., Kissinger, E., Sharma, R., Erik Magnusson, N., Puri, V., Kopchick, J.J., Jørgensen, J.O.L., and Jessen, N. (2020). The acute effects of growth hormone in adipose tissue is associated with suppression of antilipolytic signals. Phys. Rep. *8*, e14373.
40. Møller, N., Jørgensen, J.O., Schmitz, O., Møller, J., Christiansen, J., Alberti, K.G., and Orskov, H. (1990). Effects of a growth hormone pulse on total and forearm substrate fluxes in humans. Am. J. Physiol. *258*, E86–E91.
41. Ran, L., Wang, X., Mi, A., Liu, Y., Wu, J., Wang, H., Guo, M., Sun, J., Liu, B., Li, Y., et al. (2019). Loss of adipose growth hormone receptor in mice enhances local fatty acid trapping and impairs Brown adipose tissue thermogenesis. iScience *16*, 106–121.
42. Yakar, S., Setser, J., Zhao, H., Stannard, B., Haluzik, M., Glatt, V., Bouxsein, M.L., Kopchick, J.J., and LeRoith, D. (2004). Inhibition of growth hormone action improves insulin sensitivity in liver IGF-1-deficient mice. J. Clin. Invest. *113*, 96–105.
43. Yakar, S., Liu, J.L., Fernandez, A.M., Wu, Y., Schally, A.V., Frystyk, J., Chernausek, S.D., Mejia, W., and Le Roith, D. (2001). Liver-specific igf-1 gene deletion leads to muscle insulin insensitivity. Diabetes *50*, 1110–1118.
44. Mestron, A., Webb, S.M., Astorga, R., Benito, P., Catala, M., Gaztambide, S., Gomez, J.-M., Halperin, I., Lucas-Morante, T., Moreno, B., et al. (2004). Epidemiology, clinical characteristics, outcome, morbidity and mortality in acromegaly based on the Spanish Acromegaly Registry (Registro Espanol de Acromegalia, REA). Eur. J. Endocrinol. *151*, 439–446.
45. Guevara-Aguirre, J., Balasubramanian, P., Guevara-Aguirre, M., Wei, M., Madia, F., Cheng, C.-W., Hwang, D., Martin-Montalvo, A., Saavedra, J., Ingles, S., et al. (2011). Growth hormone receptor deficiency is associated with a major reduction in pro-aging signaling, cancer, and diabetes in humans. Sci. Transl. Med. *3*, 70ra13.
46. Woods, K.A., Camacho-Hübner, C., Savage, M.O., and Clark, A.J. (1996). Intrauterine growth retardation and postnatal growth failure associated with deletion of the insulin-like growth factor I gene. N. Engl. J. Med. *335*, 1363–1367.
47. Woods, K.A., Camacho-Hübner, C., Bergman, R.N., Barter, D., Clark, A.J., and Savage, M.O. (2000). Effects of insulin-like growth factor I (IGF-I) therapy on body composition and insulin resistance in IGF-I gene deletion. J. Clin. Endocrinol. Metab. *85*, 1407–1411.
48. Yamashita, H., Takenoshita, M., Sakurai, M., Bruick, R.K., Henzel, W.J., Shillinglaw, W., Arnot, D., and Uyeda, K. (2001). A glucose-responsive transcription factor that regulates carbohydrate metabolism in the liver. Proc. Natl. Acad. Sci. USA *98*, 9116–9121.
49. Ma, L., Tsatsos, N.G., and Towle, H.C. (2005). Direct role of ChREBP.Mlx in regulating hepatic glucose-responsive genes. J. Biol. Chem. *280*, 12019–12027.
50. Stoeckman, A.K., Ma, L., and Towle, H.C. (2004). Mlx is the functional heteromeric partner of the carbohydrate response element-binding protein in glucose regulation of lipogenic enzyme genes. J. Biol. Chem. *279*, 15662–15669.
51. Abdul-Wahed, A., Guilmeau, S., and Postic, C. (2017). Sweet sixteenth for ChREBP: established roles and future goals. Cell Metabol. *26*, 324–341.
52. Jois, T., Chen, W., Howard, V., Harvey, R., Youngs, K., Thalmann, C., Saha, P., Chan, L., Cowley, M.A., and Sleeman, M.W. (2017). Deletion of hepatic carbohydrate response element binding protein (ChREBP) impairs glucose homeostasis and hepatic insulin sensitivity in mice. Mol. Metabol. *6*, 1381–1394.
53. Vijayakumar, A., Aryal, P., Wen, J., Syed, I., Vazirani, R.P., Moraes-Vieira, P.M., Camporez, J.P., Gallop, M.R., Perry, R.J., Peroni, O.D., et al. (2017). Absence of carbohydrate response element binding protein in adipocytes

causes systemic insulin resistance and impairs glucose transport. Cell Rep. *21*, 1021–1035.

54. Iizuka, K., Bruick, R.K., Liang, G., Horton, J.D., and Uyeda, K. (2004). Deficiency of carbohydrate response element-binding protein (ChREBP) reduces lipogenesis as well as glycolysis. Proc. Natl. Acad. Sci. USA *101*, 7281–7286.
55. Ruth, K.S., Day, F.R., Tyrrell, J., Thompson, D.J., Wood, A.R., Mahajan, A., Beaumont, R.N., Wittemans, L., Martin, S., Busch, A.S., et al. (2020). Using human genetics to understand the disease impacts of testosterone in men and women. Nat. Med. *26*, 252–258.
56. Kanai, M., Akiyama, M., Takahashi, A., Matoba, N., Momozawa, Y., Ikeda, M., Iwata, N., Ikegawa, S., Hirata, M., Matsuda, K., et al. (2018). Genetic analysis of quantitative traits in the Japanese population links cell types to complex human diseases. Nat. Genet. *50*, 390–400.
57. Klarin, D., Damrauer, S.M., Cho, K., Sun, Y.V., Teslovich, T.M., Honerlaw, J., Gagnon, D.R., DuVall, S.L., Li, J., Peloso, G.M., et al. (2018). Genetics of blood lipids among ∼300, 000 multi-ethnic participants of the Million Veteran Program. Nat. Genet. *50*, 1514–1523.
58. Stagi, S., Lapi, E., Cecchi, C., Chiarelli, F., D’Avanzo, M.G., Seminara, S., and de Martino, M. (2014). Williams-beuren syndrome is a genetic disorder associated with impaired glucose tolerance and diabetes in childhood and adolescence: new insights from a longitudinal study. Horm. Res. Paediatr. *82*, 38–43.
59. Fry, A., Littlejohns, T.J., Sudlow, C., Doherty, N., Adamska, L., Sprosen, T., Collins, R., and Allen, N.E. (2017). Comparison of sociodemographic and health-related characteristics of UK Biobank participants with those of the general population. Am. J. Epidemiol. *186*, 1026–1034.
60. Bycroft, C., Freeman, C., Petkova, D., Band, G., Elliott, L.T., Sharp, K., Motyer, A., Vukcevic, D., Delaneau, O., O’Connell, J., et al. (2018). The UK Biobank resource with deep phenotyping and genomic data. Nature *562*, 203–209.
61. Danecek, P., Bonfield, J.K., Liddle, J., Marshall, J., Ohan, V., Pollard, M.O., Whitwham, A., Keane, T., McCarthy, S.A., Davies, R.M., and Li, H. (2021). Twelve years of SAMtools and BCFtools. GigaScience *10*, giab008. https://doi.org/10.1093/gigascience/giab008.
62. McLaren, W., Gil, L., Hunt, S.E., Riat, H.S., Ritchie, G.R.S., Thormann, A., Flicek, P., and Cunningham, F. (2016). The ensembl variant effect predictor. Genome Biol. *17*, 122.
63. Rentzsch, P., Witten, D., Cooper, G.M., Shendure, J., and Kircher, M. (2019). CADD: predicting the deleteriousness of variants throughout the human genome. Nucleic Acids Res. *47*, D886–D894.
64. Karczewski, K.J., Francioli, L.C., Tiao, G., Cummings, B.B., Alföldi, J., Wang, Q., Collins, R.L., Laricchia, K.M., Ganna, A., Birnbaum, D.P., et al. (2020). The mutational constraint spectrum quantified from variation in 141, 456 humans. Nature *581*, 434–443.
65. Eastwood, S.V., Mathur, R., Atkinson, M., Brophy, S., Sudlow, C., Flaig, R., de Lusignan, S., Allen, N., and Chaturvedi, N. (2016). Algorithms for the capture and adjudication of prevalent and incident diabetes in UK Biobank. PLoS One *11*, e0162388.
66. Seabold, S., and Perktold, J. (2010). Statsmodels: econometric and statistical modeling with Python. In Proceedings of the 9th Python in Science Conference. https://doi.org/10.25080/majora-92bf1922-011.
67. Clogg, C.C., Petkova, E., and Haritou, A. (1995). Statistical methods for comparing regression coefficients between models. Am. J. Sociol. *100*, 1261–1293. https://doi.org/10.1086/230638.
68. Ghoussaini, M., Mountjoy, E., Carmona, M., Peat, G., Schmidt, E.M., Hercules, A., Fumis, L., Miranda, A., Carvalho-Silva, D., Buniello, A., et al. (2021). Open Targets Genetics: systematic identification of trait-associated genes using large-scale genetics and functional genomics. Nucleic Acids Res. *49*, D1311–D1320.
69. Giambartolomei, C., Vukcevic, D., Schadt, E.E., Franke, L., Hingorani, A.D., Wallace, C., and Plagnol, V. (2014). Bayesian test for colocalisation between pairs of genetic association studies using summary statistics. PLoS Genet. *10*, e1004383.
70. Boughton, A.P., Welch, R.P., Flickinger, M., VandeHaar, P., Taliun, D., Abecasis, G.R., and Boehnke, M. (2021). LocusZoom.js: interactive and embeddable visualization of genetic association study results. Bioinformatics *37*, 3017–3018. https://doi.org/10.1093/bioinformatics/btab186.
71. Smith, G.D., and Ebrahim, S. (2003). “Mendelian randomization”: can genetic epidemiology contribute to understanding environmental determinants of disease? Int. J. Epidemiol. *32*, 1–22.
72. Slob, E.A.W., and Burgess, S. (2020). A comparison of robust Mendelian randomization methods using summary data. Genet. Epidemiol. *44*, 313–329.
73. Bowden, J., Del Greco M, F., Minelli, C., Davey Smith, G., Sheehan, N., and Thompson, J. (2017). A framework for the investigation of pleiotropy in two-sample summary data Mendelian randomization. Stat. Med. *36*, 1783–1802.
74. Burgess, S., Bowden, J., Fall, T., Ingelsson, E., and Thompson, S.G. (2017). Sensitivity analyses for robust causal inference from mendelian randomization analyses with multiple genetic variants. Epidemiology *28*, 30–42.
75. Bowden, J., Davey Smith, G., and Burgess, S. (2015). Mendelian randomization with invalid instruments: effect estimation and bias detection through Egger regression. Int. J. Epidemiol. *44*, 512–525.
76. Bowden, J., Davey Smith, G., Haycock, P.C., and Burgess, S. (2016). Consistent estimation in mendelian randomization with some invalid instruments using a weighted median estimator. Genet. Epidemiol. *40*, 304–314.
77. Hemani, G., Tilling, K., and Davey Smith, G. (2017). Orienting the causal relationship between imprecisely measured traits using GWAS summary data. PLoS Genet. *13*, e1007081.
78. Burgess, S., and Thompson, S.G. (2015). Multivariable Mendelian randomization: the use of pleiotropic genetic variants to estimate causal effects. Am. J. Epidemiol. *181*, 251–260.
79. Hemani, G., Zheng, J., Elsworth, B., Wade, K.H., Haberland, V., Baird, D., Laurin, C., Burgess, S., Bowden, J., Langdon, R., et al. (2018). The MR-Base platform supports systematic causal inference across the human phenome. Elife *7*, e34408. https://doi.org/10.7554/eLife.34408.

STAR★METHODS

KEY RESOURCES TABLE

REAGENT or RESOURCE	SOURCE	IDENTIFIER
Deposited data		
UK Biobank Data returns (to be submitted on publication)	UK Biobank	Application: 9905
UK Biobank Phenotypic data (detailed in Table S5)	UK Biobank	Application: 9905
UK Biobbank Whole Exome Sequence (450,000 release)	UK Biobank	Data field: 23148
T2D Knowledge Portal	https://t2d.hugeamp.org	N/A
Processed and deidentified figure data and code	This paper	https://github.com/eugenegardner/T2D_IGF1R; https://doi.org/10.5281/zenodo.7057670
Open Targets Genetics Platform	https://genetics.opentargets.org/	N/A
Software and algorithms		
BOLT-LMM	https://alkesgroup.broadinstitute.org/BOLT-LMM/BOLT-LMM_manual.html	v2.3.6
STAAR	https://github.com/xihaoli/STAAR	v0.9.6
bcftools	https://github.com/samtools/bcftools	v1.14
MRC-EPID WES Pipeline	https://github.com/mrcepid-rap/	N/A
plink/plink2	https://www.cog-genomics.org/plink/	N/A
Variant Effect Predictor	https://www.ensembl.org/info/docs/tools/vep/index.html	v104
coloc R package	https://cran.r-project.org/web/packages/coloc/index.html	v5.1.0
TwoSampleMR R package	https://mrcieu.github.io/TwoSampleMR/	v0.5.6

RESOURCE AVAILABILITY

Lead contact

Further information and requests for resources should be directed and will be fulfilled by the lead contact, John R. B. Perry (john.perry@mrc-epid.cam.ac.uk).

Materials availability

Rare variant burden testing summary statistics are included in the supplemental information of this paper. Protected UK Biobank participant data will be returned to the UK Biobank resource and be accessible via application number 9905.

Data and code availability

Code used to perform analyses and generate figures and tables for this manuscript is available on github at the following repository: https://github.com/eugenegardner/T2D_IGF1R; an unchanging version of this code at the time of publication is also available on Zenodo: https://doi.org/10.5281/zenodo.7057670. Derived variables and summary statistics generated as part of this publication have been returned to the UK Biobank and will be made available via their returns catalog under application 9905.

METHOD DETAILS

UK biobank data processing and quality control

To conduct rare variant burden analyses outlined in this publication, we queried ES data for 454,787 individuals provided by the UKBB study.[5] Individuals were excluded from further analysis if they had excess heterozygosity, autosomal variant missingness on genotyping arrays $\geq$5%, or were not included in the subset of phased samples as defined in Bycroft et al.[60] We further excluded

all study participants who were not of broadly European genetic ancestry, leaving a total of 420,162 individuals for further analysis. Additional individuals were subsequently excluded when assessing specific phenotypes due to the incomplete nature of survey data for somephenotypes (Table S5).

To perform variant quality control and annotation, we utilised the UKBB Research Analysis Platform (RAP; https://ukbiobank.dnanexus.com/). The RAP is a cloud-based compute environment which provides a central data repository for UKBB ES and phenotypic data. Using bespoke applets designed for the RAP, we performed additional quality control of ES data beyond that already documented in Backman et al.[5] Using provided population-level Variant Call Format (VCF) files, we first split and left-corrected multi-allelic variants into separate alleles using ‘bcftools norm’.[61] Next, we performed genotype-level filtering using ‘bcftools filter’ separately for Single Nucleotide Variants (SNVs) and Insertions/Deletions (InDels) using a missingness-based approach. With this approach, SNV genotypes with depth <7 and genotype quality <20 or InDel genotypes with a depth <10 and genotype quality <20 were set to missing (i.e. ./.). We further tested for an expected alternate allele contribution of 50% for heterozygous SNVs using a binomial test; SNV genotypes with a binomial test p. value $\leq 1 \times 10^{-3}$ were set to missing. Following genotype-level filtering we recalculated the proportion of individuals with a missing genotype for each variant and filtered all variants with a missingness value >50%.

We next annotated variants using the ENSEMBL Variant Effect Predictor (VEP) v104[62] with the ‘–everything’ flag and plugins for REVEL,[15] CADD,[63] and LOFTEE[64] enabled. For each variant, we prioritised a single ENSEMBL transcript based on whether or not the annotated transcript was protein-coding, MANE select v0.97, or the VEP Canonical transcript, respectively. Individual consequence for each variant was prioritised based on severity as defined by VEP. Following annotation, we grouped stop gained, frameshift, splice acceptor, and splice donor variants into a single Protein Truncating Variant (PTV) category. Missense and synonymous variant consequences are identical to those defined by VEP. Only autosomal or chrX variants within ENSEMBL protein-coding transcripts and within transcripts included on the UKBB ES assay were retained for subsequent burden testing.

Exome-wide association analyses in the UK biobank

To perform rare variant burden tests using filtered and annotated ES data, we employed a custom implementation of BOLT-LMM v2.3.6[17] for the RAP. BOLT-LMM expects two primary inputs: i) a set of genotypes with minor allele count >100 derived from genotyping arrays to construct a null model and ii) a larger set of imputed variants to perform association tests. For the former, we queried genotyping data available on the RAP and restricted to an identical set of individuals used for rare variant association tests. For the latter, and as BOLT-LMM expects imputed genotyping data as input rather than per-gene carrier status, we created dummy genotype files where each variant represents one gene and individuals with a qualifying variant within that gene are coded as heterozygous, regardless of the number of variants that individual has in that gene. To test a range of variant annotation categories across the allele frequency spectrum, we created dummy genotype files for minor allele frequency <0.1% and singleton high confidence PTVs as defined by LOFTEE, missense variants with REVEL $\geq$0.5, missense variants with REVEL $\geq$0.7, and synonymous variants. For each phenotype tested, BOLT-LMM was then run with default parameters other than the inclusion of the ‘lmmInfOnly’ flag. When exploring the role of rare variants in the IGF-1/GH axis and to incorporate less deleterious missense variants, we also used an additional set of variant annotations which combined missense variants with CADD $\geq$25 and high confidence PTVs (i.e. Damaging; Table S3). To derive association statistics for individual variants, we also provided all 26,657,229 individual markers regardless of filtering status as input to BOLT-LMM. All tested phenotypes were run as continuous traits corrected by age, age,[2] sex, the first ten genetic principal components as calculated in Bycroft et al.,[60] and study participant ES batch as a categorical covariate (either 50 k, 200 k, or 450 k). For phenotype definitions used in this study, including our T2D definition adapted from Eastwood et al.,[65] please refer to Table S5. Only the first instance (initial visit) was used for generating all phenotype definitions unless specifically noted in Table S5.

To provide an orthogonal approach to validate our BOLT-LMM results, we also performed per-gene burden tests with STAAR[16] and a generalised linear model as implemented in the python package ‘statsmodels’.[66] To run STAAR, we created a custom Python and R workflow on the RAP. VCF files were first converted into a sparse matrix suitable for use with the R package ‘Matrix’ using ‘bcftools query’. Using the ‘STAAR’ R package, we first ran a null model with identical coefficients to BOLT-LMM and a sparse relatedness matrix with a relatedness coefficient cutoff of 0.125 as described by Bycroft et al.[60] We next used the function ‘STAAR’ to test all protein-coding transcripts as outlined above. To run generalised linear models, we used a three step process. First, we ran a null model with all dependent variables as continuous traits, corrected for control covariates identical to those included in BOLT-LMM. Next, using the residuals of this null model, we performed initial regressions on carrier status to obtain a preliminary p. value. Finally, for individual genes that passed a lenient p. value threshold of $<1 \times 10^{-4}$, we recalculated a full model to obtain exact test statistics with family set to ‘binomial’ or ‘gaussian’ if the trait was binary or continuous, respectively. Generalised linear models utilised identical input to BOLT-LMM converted to a sparse matrix.

To conduct heterogeneity tests between two calculated odds ratios, we implemented a Z test as sample sizes were sufficiently large.[67] This approach was used to determine if missense variants within the *IGF1R* protein kinase domain have a stronger effect on T2D risk than those elsewhere in *IGF1R*. A Z-score was calculated by dividing the difference in the coefficients by the standard error of the difference, as shown below:

$Z = (\text{Beta from model 1}) - (\text{Beta from model 2})/((\text{SE of Beta from model 1})^2 + (\text{SE of Beta from model 2})^2)^{1/2}$

P-values were estimated for Z scores from a normal distribution.

Common variant GWAS lookups

Common variant associations at the identified genes were queried using the T2D Knowledge Portal (https://t2d.hugeamp.org) and the Open Targets Genetics platform (https://genetics.opentargets.org/).[68] Trait associations from the T2D Knowledge Portal are presented in Table S2 and were only included if the paired gene was assigned as the nearest gene to the association signal as a crude proxy for causality. Accompanying HuGE scores were extracted for the highest-scoring glycaemic common variants associations. Locus2Gene scores based on data from Vujkovic et al.[2] were extracted from the Open Targets Genetics platform and are presented in Table S2. For the *IGF1R* locus follow-up, we used sentinel SNP information for Vujkovic et al.[2] and summary statistics from the recent fasting glucose MAGIC meta-analysis[21] and circulating IGF-1 levels GWAS. eQTL data was accessed through GTEx v8[22]. Effect estimates in the text have been aligned toward the T2D/glucose increasing alleles, using LD information from LDlink.[22] Colocalization between the IGF-1 and fasting glucose GWASs was ascertained using coloc v5.1.0[69] (Figure 3), where posterior probability for H3 (i.e. PP3) is the probability of two independent causal variants, while H4 is the probability of a single, shared causal variant. For this, variants within a 500 kb window of IGF1R that were common between the two studies were used. Regions in Figure 3 were plotted using LocusZoom.[70]

Mendelian randomisation using IGF-1 levels

To examine the likelihood of a causal effect of IGF-1 on the risk of T2D, we applied Mendelian randomisation (MR) analysis. In this approach, genetic variants that are significantly associated with an exposure of interest are used as instrumental variables (IVs) to test the causality of that exposure on the outcome of interest. For a genetic variant to be a reliable instrument, the following assumptions should be met: (1) the genetic instrument is associated with the exposure of interest, (2) the genetic instrument should not be associated with any other competing risk factor that is a confounder, and (3) the genetic instrument should not be associated with the outcome, except via the causal pathway that includes the exposure of interest.[71] As IVs, we used the 831 IGF-1 genome-wide significant signals reported in a recent GWAS on IGF-1.[24] As our outcome data, we selected the largest publicly available independent T2D dataset available in 893,130 European genetic ancestry individuals (9% cases) from Mahajan et al.[31] If a signal was not present in the outcome GWAS, we searched the UKBB white European dataset for proxies (within 1 Mb and $r^2 > 0.5$) and chose the variant with the highest r^2 value, which left 784 independent markers for MR analysis. Genotypes at all variants were aligned to designate the IGF-1-increasing alleles as the effect alleles.

To conduct our MR analysis, we used the inverse-variance weighted (IVW) model as the primary model as it offers the most statistical power[72]; however, as it does not correct for heterogeneity in outcome risk estimates between individual variants,[73] we applied a number of sensitivity MR methods that better account for heterogeneity.[74] These include an Egger analysis to identify and correct for unbalanced heterogeneity ('horizontal pleiotropy'), indicated by a significant Egger intercept ($p<0.05$),[75] and weighted median (WM) and penalised weighted median (PWM) models to correct for balanced heterogeneity.[76] In addition, we introduced the radial method to exclude variants from each model in cases where they are recognized as outliers, as well as Steiger filtering to assess for potential reverse causality (i.e. variants with stronger association with the outcome than with the exposure).[77] As previous work on IGF-1 showed a strong association with height, and to a lesser extent BMI,[30] we also used multivariable MR analysis[78] to estimate the direct effect of IGF-1 levels on T2D not mediated by BMI or height by adjusting for their effects as covariates using queried phenotype data for UKBB participants (Tables S4 and S5). In order to examine the individual level effect of IGF1 and IGF1R loci on T2D, BMI, childhood and adult height, we performed the variant-specific lookups as well as calculated the Wald ratio using the R package 'TwoSampleMR'.[79] All results presented in the main text are expressed in standard deviations of IGF-1 levels (one S.D. is equivalent to ~5.5 nmol/L in UKBB). Values available in Table S4 are raw data, per unit IGF-1.

Cell Genomics, Volume *2*

Supplemental information

Damaging missense variants in *IGF1R* implicate a role for IGF-1 resistance in the etiology of type 2 diabetes

Eugene J. Gardner, Katherine A. Kentistou, Stasa Stankovic, Samuel Lockhart, Eleanor Wheeler, Felix R. Day, Nicola D. Kerrison, Nicholas J. Wareham, Claudia Langenberg, Stephen O'Rahilly, Ken K. Ong, and John R.B. Perry

Supplementary Materials for

Damaging missense variants in *IGF1R* implicate a role for IGF-1 resistance in the aetiology of type 2 diabetes

Eugene J. Gardner[@], Katherine Kentistou, Stasa Stankovic, Samuel Lockhart, Eleanor Wheeler, Felix R. Day, Nicola D. Kerrison, Nicholas J. Wareham, Claudia Langenberg, Stephen O'Rahilly*, Ken K. Ong*, John R. B. Perry*[,@]

* These authors jointly supervised this work
[@] Corresponding authors: eugene.gardner@mrc-epid.cam.ac.uk; john.perry@mrc-epid.cam.ac.uk

Supplementary Figures

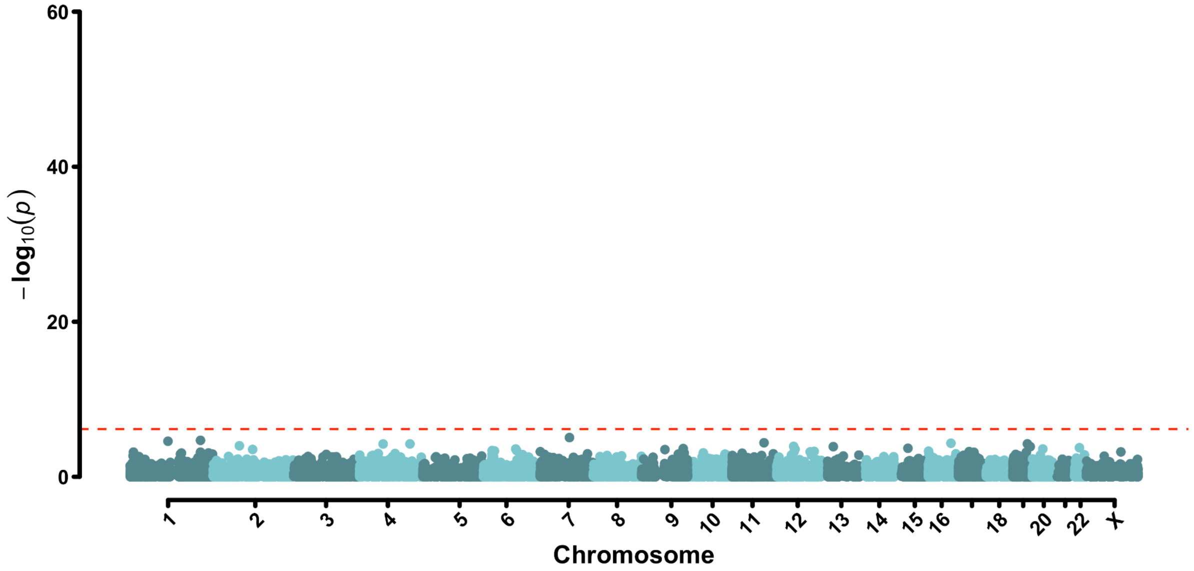

Supplementary Figure S1. Exome-wide association results for synonymous variants, related to Figure 1.

Plotted are per-gene burden results when only considering synonymous variants. The red line indicates our exome-wide significant p value after Bonferroni correction of 6.9×10^{-7}.

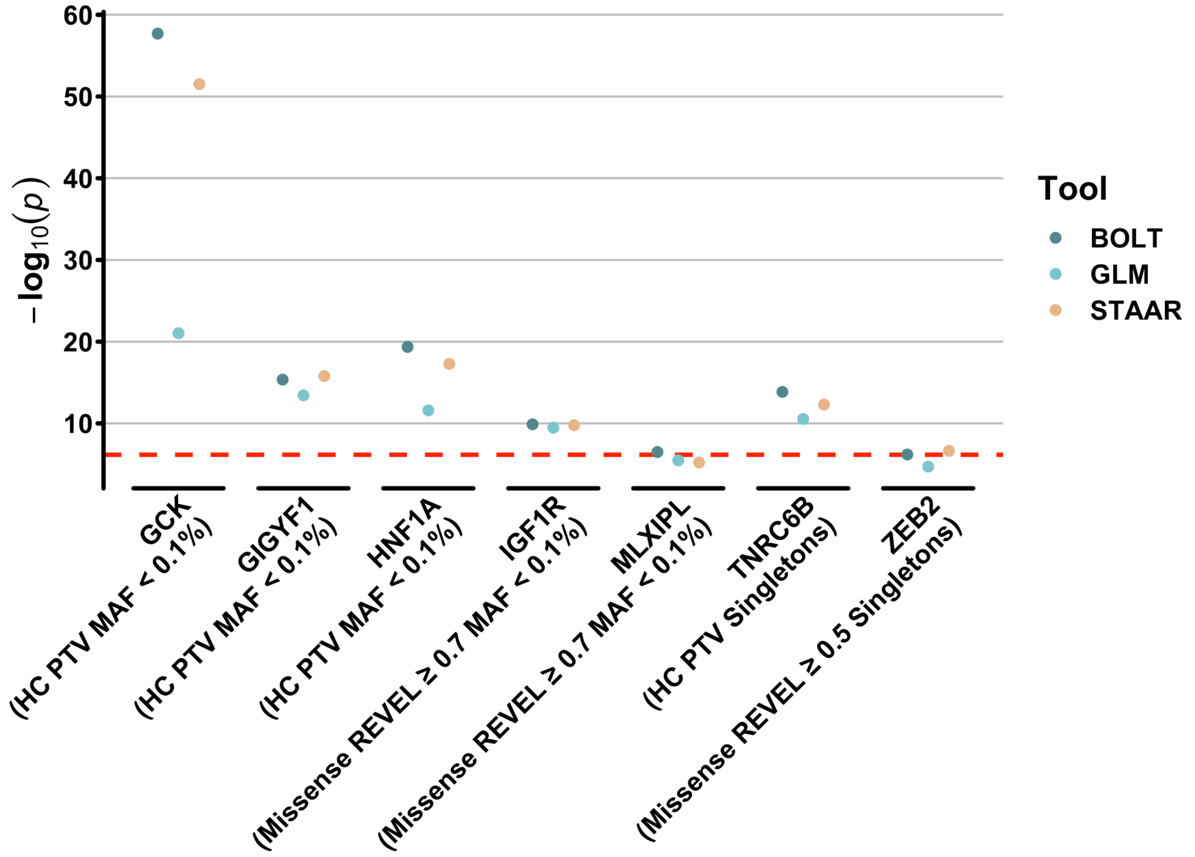

Supplementary Figure S2. Exome-wide association results for additional methods, related to Figure 1.

Displayed are association results for both our primary approach (BOLT) as well as two other orthogonal approaches (generalised linear models (GLM) and STAAR). -$\log_{10}$(p values) for each tool are plotted only for all genes identified as exome-wide significant with BOLT.

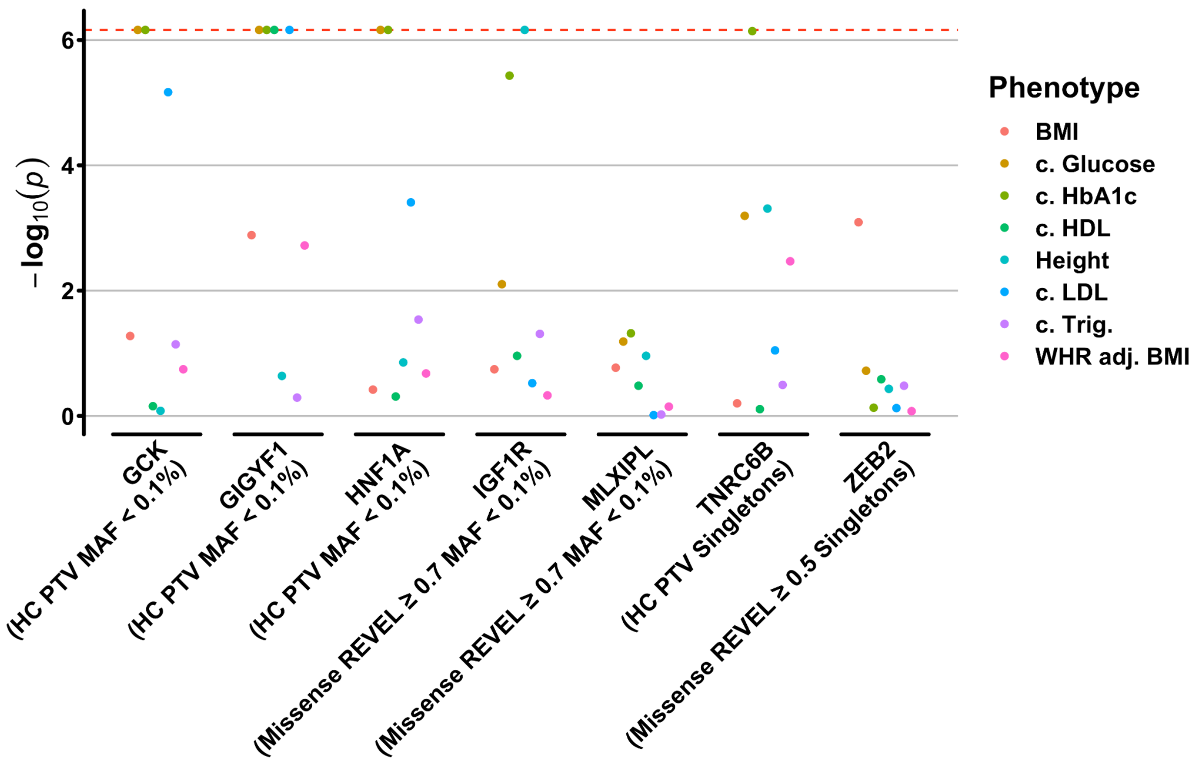

Supplementary Figure S3. Additional phenotypes associated with T2D or the GH-IGF1 pathway, related to Figure 1.

Shown are associations between rare variant burden and eight additional phenotypes with known associations to type 2 diabetes or the GH-IGF1 signalling pathway. Plotted are $-\log_{10}$ p. values for each trait derived from the mask-MAF cutoff most significantly associated with T2D (Main Text Figure 1; Supplementary Table 1). $-\log_{10}$ p. values are capped at exome wide significance ($-\log_{10} p = 6.16$) to enable comparison of nominally associated signals. ‘c.’, ‘trig.’, and ‘adj.’ in the figure legend stand for ‘circulating’, ‘triglycerides’, and ‘adjusted’, respectively.

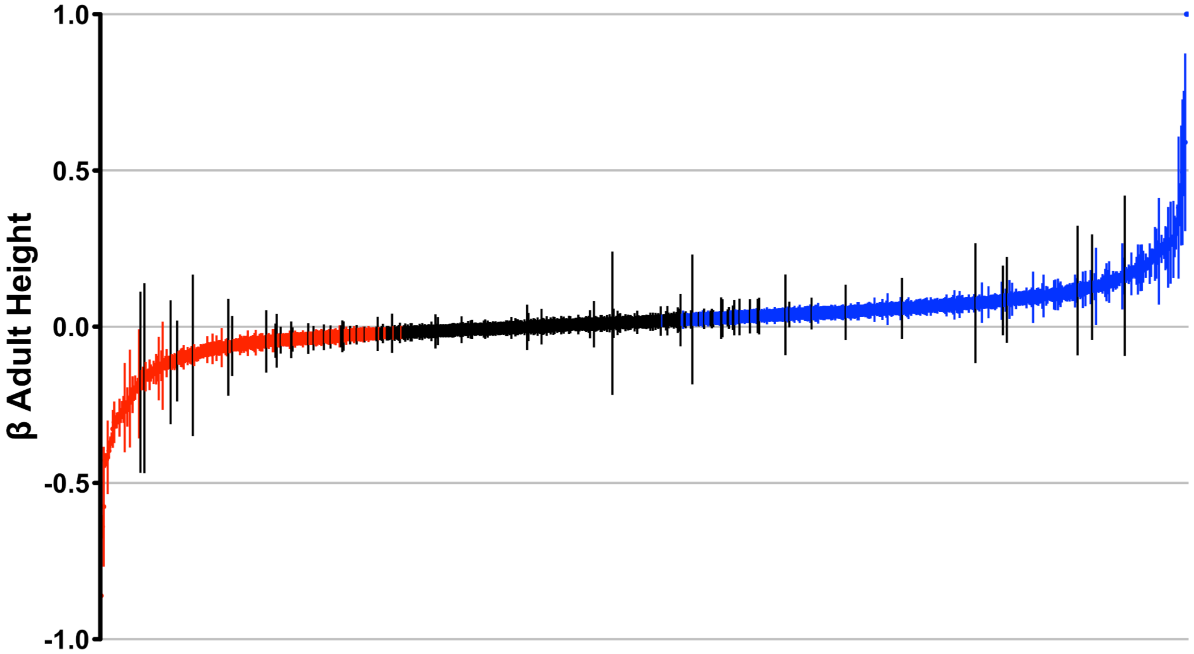

Supplementary Figure S4. Heterogeneity with height of SNPs associated with IGF-1, related to STAR Methods.
Shown are Mendelian randomisation results for 784 independent genetic signals with a known association with circulating IGF-1 levels. SNPs are ordered on the x-axis by their β value for adult height. SNPs in red have a significantly negative association with adult height while SNPs in blue have a significantly positive association with adult height (y-axis). Note that the y-axis has been capped at -1 and +1; two SNPs above this cap in the upper-right hand corner have β's of 1.6 [1.3-1.8] and 1.9 [1.6-2.2]. Error bars represent the 95% confidence interval.
