# Supplementary material for: Damaging missense variants in IGF1R implicate a role for IGF-1 resistance in the etiology of type 2 diabetes
Source: Cell Genom. 2022 Nov 7;2(12):100208. doi: 10.1016/j.xgen.2022.100208 (PMC9750938; doi:10.1016/j.xgen.2022.100208)
Supplement: Document S2. Transparent peer review records for Gardner et al [file mmc7.pdf]

Cell Genomics Transparent Peer Review Record

Damaging missense variants in *IGF1R* implicate a role for IGF-1 resistance in the aetiology of type 2 diabetes

Eugene J. Gardner[@,1,a], Katherine A. Kentistou[1], Stasa Stankovic[1], Samuel Lockhart[2], Eleanor Wheeler[1], Felix R. Day[1], Nicola D. Kerrison[1], Nicholas J. Wareham[1], Claudia Langenberg[1,4], Stephen O'Rahilly*[,2,3], Ken K. Ong*[1,5], John R. B. Perry*[,@,1,6]

Summary

Initial submission: Received : 4/14/2022

Scientific editor: Emily Marcinkevicius, Rita Gemayel, Laura M. Zahn

First round of review: Number of reviewers: 2
Revision invited : 7/6/2022
Revision received : 8/12/2022

Second round of review: Number of reviewers: 2
Accepted : 10/7/2022

Data freely available: Uses UKBB and requires request for access

Code freely available: Yes

This transparent peer review record is not systematically proofread, type-set, or edited. Special characters, formatting, and equations may fail to render properly. Standard procedural text within the editor's letters has been deleted for the sake of brevity, but all official correspondence specific to the manuscript has been preserved.

CellPress

Referees' reports, first round of review

Reviewer #1: In this manuscript, E. Gardner, J. Perry and colleagues from the Cambridge group present a well powered genome-wide, gene-based analysis for type 2 diabetes (T2D) based on whole-exome sequences in 418,436 UK Biobank participants of European descent, of whom 32,374 had T2D. Their dataset covered 18,691 genes, and results were predicated on meeting exome-wide significance ($P<6.9\times10-7$), concentrating on rare, damaging missense variants. Their findings include "positive controls" at the known diabetes genes GCK, GIGYF1, HNF1A and TNRC6B, but they also identify novel associations for ZEB2 (large odds ratio [OR] of 5.5 but only 31 observations, so wide 95% confidence intervals), IGF1R and MLXIPL. They focus on IGF1R based on the known biology and high relevance: they document an association of the same damaging variants with short stature despite higher circulating IGF-1 levels, which is most consistent with IGF-1 resistance conferred by receptor mutations leading to compensatory rises in circulating IGF-1. In support of this notion, a Mendelian randomization analysis of 784 common IGF-1-raising variants confirmed the previously observed relationship between higher genetically determined IGF-1 levels and higher T2D risk, but also documented significant heterogeneity in the effect of this genetic instrument on both height and T2D, indicating that variants that primarily raise IGF-1 levels and enhance downstream signalling might have different effects from those that confer IGF-1 resistance and thereby induce a compensatory increase in IGF-1 levels, but with reduced downstream signalling. The biological relevance of the other novel finding with a robust number of observations (MLXIPLI) is also highlighted, as this gene encodes the carbohydrate response element binding protein.

This well written and compelling paper has a number of strengths:

1. A focus on coding variation that allows for straightforward gene-based, genome-wide analyses
2. A clever filter of variants focusing on those most likely to be damaging
3. A well-powered dataset with >25,000 T2D cases and many more controls
4. A deliberate effort to limit population heterogeneity as a way to facilitate interpretation and avoid potential artefacts when examining rare variation
5. State-of-the-art tools for genomic analyses
6. A number of positive controls that confirm the adequacy of the approach
7. A number of novel interesting findings, including a highly relevant pathway via IGF-1 signaling that is most consistent with IGF-1 resistance leading to shorter stature and higher T2D risk, with multiple lines of evidence supporting this model and suggesting further functional experiments to help establish a dominant negative mode of transmission
8. Broad leverage of available genomic resources to test their leading hypothesis
9. Translational implications regarding modulation of the GH axis and T2D risk

I only have a number of minor suggestions offered for the authors' consideration to enhance clarity in the paper:

1. Line 113: prefer people-first language, replace "T2D patients" with "participants with T2D"
2. Figure 2: most readers are used to straight OR rather than log odds, and thus the IGF1R triangle below 1.0 seems confusing. Would consider replacing the Y axis scale with straight OR and truncating it if necessary for the strong-effect findings
3. In the same figure, "Type II Diabetes" should be "Type 2 Diabetes"
4. For the data in Figure 3, formal colocalization analyses of fasting glucose and IGF-1 levels should be conducted, if adequate datasets are available
5. Lines 169-170: the statement that the risk of T2D was higher for carriers of variants in the kinase domain compared to carriers of variants outside the domain implies that two ORs are significantly different from

each other. However, the 95% confidence intervals do overlap a bit, so in the absence of a formal statistical test (e.g. for heterogeneity) the statement should be qualified accordingly

6. The authors note substantial heterogeneity on the effect of the 784 IGF-1 level-raising variants on both T2D and adult height, and in the Discussion they propose that common variants be evaluated for effects on a biological readout before they are included in genetic instruments. In the major hypothesis posed by this paper, reduced IGF-1 signalling induced by IGF1R damaging variants causes both compensatory higher IGF-1 levels and higher T2D risk; the corollary is that increased IGF-1 signalling (potentially caused by primary IGF-1-raising variants) should both increase height and lower T2D risk. Could the authors test this by creating two separate genetic instruments for IGF-1 levels, one composed of alleles that raise both IGF-1 levels and height and one composed of alleles that raise IGF-1 levels but reduce height, and see if they have the expected effects on T2D? It is possible that significant heterogeneity may still be present in the former of the two instruments, as this may include variants that raise IGF-1 via both GH-dependent and independent mechanisms, in which case their effect on T2D might be neutralized.

7. The statement in Lines 197-198 of the Results "damaging rare variants that disrupt IGF-1 bioactivity, but not those that alter GH secretion or signalling, appear to increase T2D risk" seems to contradict the statement in Lines 271-272 of the Discussion "the IGF1R-independent effects of GH are likely to have a deleterious effect on systemic glucose metabolism".

8. Line 252 - I find the expression "may play a role" overused and underinformative: it seems better to actually describe the role played, if such information exists (e.g. "may by transducing IGF-1 signalling condition the development and growth of several tissues central to glucose metabolism…")

9. Lines 398, 400 and elsewhere: please be consistent with US or British spelling

Reviewer #2: Gardner et al. have conducted an Exome-Wide gene burden analysis in 418,436 (n=32,374 T2D cases) individuals sequenced by the UK Biobank study to assess the impact of rare genetic variants on T2D risk. Overall, the statistical analysis method is adequate; manuscript is well written, and results are interesting. However, following issues should be addressed.

1. It is still very puzzling why PTVs group in the IGF1R gene had no T2D associations, considering these were most damaging variants, although authors mentioned that it's possibly due to nonsense-mediated decay. But authors also stated that PTVs in IGF1R had effects on several growth measures in other studies, but not for damaging missense variants.

2. Authors stated that loss of function mutations in IGF1R are likely to result in compensatory increases in GH secretion which could have a profound effect on insulin resistance. Have authors also investigated a likely causal role of GH by Mendelian randomization analyses?

3. IGF1R can also bind to insulin and IGF-2. Have carriers of damaging missense variants within IGF1R also had altered circulating levels of insulin and IGF-2?

4. Have authors observed the sex-dependent association of damaging IGF1R variants withT2D?

5. The number of SNP should be indicated in the burden analysis of IGF1R?

Authors’ response to the first round of review

We are very grateful to the reviewers for their constructive comments and positive response to our work. We have responded to all comments below in red.

Reviewer #1:

In this manuscript, E. Gardner, J. Perry and colleagues from the Cambridge group present a well powered genome-wide, gene-based analysis for type 2 diabetes (T2D) based on whole-exome sequences in 418,436 UK Biobank participants of European descent, of whom 32,374 had T2D. Their dataset covered 18,691 genes, and results were predicated on meeting exome-wide significance (P<6.9´10-7), concentrating on rare, damaging missense variants. Their findings include "positive controls" at the known diabetes genes GCK, GIGYF1, HNF1A and TNRC6B, but they also identify novel associations for ZEB2 (large odds ratio [OR] of 5.5 but only 31 observations, so wide 95% confidence intervals), IGF1R and MLXIPL. They focus on IGF1R based on the known biology and high relevance: they document an association of the same damaging variants with short stature despite higher circulating IGF-1 levels, which is most consistent with IGF-1 resistance conferred by receptor mutations leading to compensatory rises in circulating IGF-1. In support of this notion, a Mendelian randomization analysis of 784 common IGF-1-raising variants confirmed the previously observed relationship between higher genetically determined IGF-1 levels and higher T2D risk, but also documented significant heterogeneity in the effect of this genetic instrument on both height and T2D, indicating that variants that primarily raise IGF-1 levels and enhance downstream signalling might have different effects from those that confer IGF-1 resistance and thereby induce a compensatory increase in IGF-1 levels, but with reduced downstream signalling. The biological relevance of the other novel finding with a robust number of observations (MLXIPLI) is also highlighted, as this gene encodes the carbohydrate response element binding protein.

This well written and compelling paper has a number of strengths:

1. A focus on coding variation that allows for straightforward gene-based, genome-wide analyses
2. A clever filter of variants focusing on those most likely to be damaging
3. A well-powered dataset with >25,000 T2D cases and many more controls
4. A deliberate effort to limit population heterogeneity as a way to facilitate interpretation and avoid potential artefacts when examining rare variation
5. State-of-the-art tools for genomic analyses
6. A number of positive controls that confirm the adequacy of the approach
7. A number of novel interesting findings, including a highly relevant pathway via IGF-1 signaling that is most consistent with IGF-1 resistance leading to shorter stature and higher T2D risk, with multiple lines of evidence supporting this model and suggesting further functional experiments to help establish a dominant negative mode of transmission
8. Broad leverage of available genomic resources to test their leading hypothesis
9. Translational implications regarding modulation of the GH axis and T2D risk

I only have a number of minor suggestions offered for the authors' consideration to enhance clarity in the paper:

1. Line 113: prefer people-first language, replace "T2D patients" with "participants with T2D"

Response to Reviewers

We agree and have made the requested change.

2. Figure 2: most readers are used to straight OR rather than log odds, and thus the IGF1R triangle below 1.0 seems confusing. Would consider replacing the Y axis scale with straight OR and truncating it if necessary for the strong-effect findings

We have made the requested change. We have opted to use an arrow, rather than a broken y-axis, to show the association of GCK with T2D risk for figure clarity.

3. In the same figure, "Type II Diabetes" should be "Type 2 Diabetes"

We have made the requested change.

4. For the data in Figure 3, formal colocalization analyses of fasting glucose and IGF-1 levels should be conducted, if adequate datasets are available
We have conducted a more formal colocalization analysis as requested using the 'coloc' software package (Giambartolomei et al., PLoS Genetics, 2014). Unfortunately colocalisation analyses could not confirm these effects were driven by the same signals, likely because of multiple secondary signals for IGF1 levels, which in light of the rare variant analyses, highlights the limitations of common variant approaches.

5. Lines 169-170: the statement that the risk of T2D was higher for carriers of variants in the kinase domain compared to carriers of variants outside the domain implies that two ORs are significantly different from each other. However, the 95% confidence intervals do overlap a bit, so in the absence of a formal statistical test (e.g. for heterogeneity) the statement should be qualified accordingly
We appreciate this comment and have now conducted a heterogeneity test to determine if the effect sizes inside and out of the IGF1R protein kinase domain are significantly different from each other. As indicated by the overlapping confidence intervals, the difference in ORs between carriers of missense variants inside and outside of the IGF1R protein kinase domain was not statistically significant. We have added this analysis and caveated our conclusions in the main text of the revised manuscript.

6. The authors note substantial heterogeneity on the effect of the 784 IGF-1 level-raising variants on both T2D and adult height, and in the Discussion they propose that common variants be evaluated for effects on a biological readout before they are included in genetic instruments. In the major hypothesis posed by this paper, reduced IGF-1 signalling induced by IGF1R damaging variants causes both compensatory higher IGF-1 levels and higher T2D risk; the corollary is that increased IGF-1 signalling (potentially caused by primary IGF-1-raising variants) should both increase height and lower T2D risk. Could the authors test this by creating two separate genetic instruments for IGF-1 levels, one composed of allels that raise both IGF-1 levels and height and one composed of alleles that raise IGF-1 levels but reduce height, and see if they have the expected effects on T2D? It is possible that significant heterogeneity may still be present in the former of the two instruments, as this may include variants that raise IGF-1 via both GH-dependent and independent mechanisms, in which case their effect on T2D might be neutralized.
We agree that we hoped to be able to generalise the current findings of the individual IGF-1 associated SNPs at IGF1 and IGF1R, by creating separate genetic instruments for higher IGF-1 levels, one composed of alleles that raise height and one that reduce height. However, this approach fails to clearly distinguish different effects on T2D:

- IGF-1-increasing+Height-decreasing alleles: OR=1.043 (1.011-1.076)
- IGF-1-increasing+Height-increasing alleles: OR=1.037 (1.022-1.057)

We aim to pursue this question by stratifying individual IGF-1-associated SNPs by additional 'intermediate traits' that capture the different plausible biological pathways that lead to raised circulating IGF-1 levels (a recent paper suggested there are at least six: Wang et al. Int J Epidemiol 2022 PMID 35656699) but we consider that such methodological work on the validity of Mendelian randomisation is beyond the remit of the current paper.

7. The statement in Lines 197-198 of the Results "damaging rare variants that disrupt IGF-1 bioactivity, but not those that alter GH secretion or signalling, appear to increase T2D risk" seems to contradict the statement in Lines 271-272 of the Discussion "the IGF1R-independent effects of GH are likely to have a deleterious effect on systemic glucose metabolism".

The statement in Lines 197-198 is an accurate description of the Results, but we agree that the statement in Lines 271-272 of the Discussion is apparently contradictory and have clarified this to “the lipolytic effects of GH are likely to have a deleterious effect on systemic glucose metabolism".

8. Line 252 - I find the expression "may play a role" overused and underinformative: it seems better to actually describe the role played, if such information exists (e.g. "may by transducing IGF-1 signalling condition the development and growth of several tissues central to glucose metabolism…")
We have modified the text to reflect the reviewer’s suggestion by using unambiguous language.

9. Lines 398, 400 and elsewhere: please be consistent with US or British spelling
We have done a thorough edit for inconsistencies in British versus US spelling.

Reviewer #2:
Gardner et al. have conducted an Exome-Wide gene burden analysis in 418,436 (n=32,374 T2D cases) individuals sequenced by the UK Biobank study to assess the impact of rare genetic variants on T2D risk. Overall, the statistical analysis method is adequate; manuscript is well written, and results are interesting. However, following issues should be addressed.

1. It is still very puzzling why PTVs group in the IGF1R gene had no T2D associations, considering these were most damaging variants, although authors mentioned that it's possibly due to nonsense-mediated decay. But authors also stated that PTVs in IGF1R had effects on several growth measures in other studies, but not for damaging missense variants.
We agree that it may be surprising that PTVs show no association with T2D risk, particularly in light of the strong T2D association for missense variants. As we suggest in the manuscript, our hypothesis is that missense variants create hypomorph IGF1R receptors. By contrast heterozygous PTVs lead to a lower dosage of normal IGF1R on the cell membrane. Consequently, the apparent contrasting effects of heterozygous IGF1R PTVs on T2D and growth traits might reflect the dose-response relationship (and redundancy) between IGF signalling and each trait. Additional wet-lab experiments beyond the scope of this manuscript will be needed to elucidate the different mechanisms associated with IGF1R loss of function versus diminished function. We believe that we have appropriately caveated our conclusions in the main text of the manuscript.
2. Authors stated that loss of function mutations in IGF1R are likely to result in compensatory increases in GH secretion which could have a profound effect on insulin resistance. Have authors also investigated a likely causal role of GH by Mendelian randomization analyses?
We agree with the reviewer that this would be extremely interesting to explore. Unfortunately, GH levels are very difficult to assess in population studies and no large-scale GWASs have been performed to date.

3. IGF1R can also bind to insulin and IGF-2. Have carriers of damaging missense variants within IGF1R also had altered circulating levels of insulin and IGF-2?
Unfortunately, neither insulin nor IGF-2 levels were measured by the UK Biobank. As such, we are unable to directly assess if missense variation within IGF1R could lead to insulin/IGF-2 resistance.

4. Have authors observed the sex-dependent association of damaging IGF1R variants with T2D?
We have now conducted sex-specific analyses at the IGF1R locus and did not identify any significant difference in effect between men and women (heterogeneity p=0.46).

5. The number of SNP should be indicated in the burden analysis of IGF1R?
We have added this value to Supplementary Table 1 for all gene/variant combinations significantly associated with increased T2D risk.

Referees' report, second round of review

Reviewer #1: The authors have been responsive to the review process.

Reviewer #2: Authors have addressed my comments.

Authors' response to the second round of review

We appreciate the reviewers constructive criticism and time necessary to review our manuscript.
